# High quality genome assembly of the anhydrobiotic midge provides insights on a single chromosome-based emergence of extreme desiccation tolerance

**DOI:** 10.1093/nargab/lqac029

**Published:** 2022-04-05

**Authors:** Yuki Yoshida, Nurislam Shaikhutdinov, Olga Kozlova, Masayoshi Itoh, Michihira Tagami, Mitsuyoshi Murata, Hiromi Nishiyori-Sueki, Miki Kojima-Ishiyama, Shohei Noma, Alexander Cherkasov, Guzel Gazizova, Aigul Nasibullina, Ruslan Deviatiiarov, Elena Shagimardanova, Alina Ryabova, Katsushi Yamaguchi, Takahiro Bino, Shuji Shigenobu, Shoko Tokumoto, Yugo Miyata, Richard Cornette, Takahiro G Yamada, Akira Funahashi, Masaru Tomita, Oleg Gusev, Takahiro Kikawada

**Affiliations:** Institute for Advanced Biosciences, Keio University, Tsuruoka, Yamagata 997-0035, Japan; Graduate School of Media and Governance, Systems Biology Program, Keio University, Fujisawa, Kanagawa 252-0882, Japan; Regulatory Genomics Research Center, Institute of Fundamental Medicine and Biology, Kazan Federal University, Kazan 420012, Russian Federation; Center of Life Sciences, Skolkovo Institute of Science and Technology, Moscow, 21205, Russian Federation; Regulatory Genomics Research Center, Institute of Fundamental Medicine and Biology, Kazan Federal University, Kazan 420012, Russian Federation; Preventive Medicine & Diagnosis Innovation Program (PMI), RIKEN, Wako, Saitama 351-0198, Japan; Center for Integrative Medical Sciences, RIKEN, Yokohama, Kanagawa 230-0045, Japan; Center for Integrative Medical Sciences, RIKEN, Yokohama, Kanagawa 230-0045, Japan; Center for Integrative Medical Sciences, RIKEN, Yokohama, Kanagawa 230-0045, Japan; Center for Integrative Medical Sciences, RIKEN, Yokohama, Kanagawa 230-0045, Japan; Center for Integrative Medical Sciences, RIKEN, Yokohama, Kanagawa 230-0045, Japan; Center for Integrative Medical Sciences, RIKEN, Yokohama, Kanagawa 230-0045, Japan; Regulatory Genomics Research Center, Institute of Fundamental Medicine and Biology, Kazan Federal University, Kazan 420012, Russian Federation; Regulatory Genomics Research Center, Institute of Fundamental Medicine and Biology, Kazan Federal University, Kazan 420012, Russian Federation; Regulatory Genomics Research Center, Institute of Fundamental Medicine and Biology, Kazan Federal University, Kazan 420012, Russian Federation; Regulatory Genomics Research Center, Institute of Fundamental Medicine and Biology, Kazan Federal University, Kazan 420012, Russian Federation; Regulatory Genomics Research Center, Institute of Fundamental Medicine and Biology, Kazan Federal University, Kazan 420012, Russian Federation; Regulatory Genomics Research Center, Institute of Fundamental Medicine and Biology, Kazan Federal University, Kazan 420012, Russian Federation; Functional Genomics Facility, National Institute for Basic Biology, Okazaki, Aichi 444-8585, Japan; Functional Genomics Facility, National Institute for Basic Biology, Okazaki, Aichi 444-8585, Japan; Functional Genomics Facility, National Institute for Basic Biology, Okazaki, Aichi 444-8585, Japan; Department of Integrated Biosciences, Graduate School of Frontier Sciences, The University of Tokyo, Kashiwa, Chiba 277-8562, Japan; Institute of Agrobiological Sciences, National Agriculture and Food Research Organization (NARO), Tsukuba, Ibaraki 305-8634, Japan; Institute of Agrobiological Sciences, National Agriculture and Food Research Organization (NARO), Tsukuba, Ibaraki 305-8634, Japan; Department of Biosciences and Informatics, Keio University, Yokohama, Kanagawa 223-8522, Japan; Department of Biosciences and Informatics, Keio University, Yokohama, Kanagawa 223-8522, Japan; Institute for Advanced Biosciences, Keio University, Tsuruoka, Yamagata 997-0035, Japan; Graduate School of Media and Governance, Systems Biology Program, Keio University, Fujisawa, Kanagawa 252-0882, Japan; Faculty of Environment and Information studies, Keio University, Fujisawa, Kanagawa 252-0882, Japan; Regulatory Genomics Research Center, Institute of Fundamental Medicine and Biology, Kazan Federal University, Kazan 420012, Russian Federation; Center for Integrative Medical Sciences, RIKEN, Yokohama, Kanagawa 230-0045, Japan; Department of Regulatory Transcriptomics for Medical Genetic Diagnostics, Graduate School of Medicine, Juntendo University, Tokyo 113-8421, Japan; Department of Integrated Biosciences, Graduate School of Frontier Sciences, The University of Tokyo, Kashiwa, Chiba 277-8562, Japan; Institute of Agrobiological Sciences, National Agriculture and Food Research Organization (NARO), Tsukuba, Ibaraki 305-8634, Japan

## Abstract

Non-biting midges (Chironomidae) are known to inhabit a wide range of environments, and certain species can tolerate extreme conditions, where the rest of insects cannot survive. In particular, the sleeping chironomid *Polypedilum vanderplanki* is known for the remarkable ability of its larvae to withstand almost complete desiccation by entering a state called anhydrobiosis. Chromosome numbers in chironomids are higher than in other dipterans and this extra genomic resource might facilitate rapid adaptation to novel environments. We used improved sequencing strategies to assemble a chromosome-level genome sequence for *P. vanderplanki* for deep comparative analysis of genomic location of genes associated with desiccation tolerance. Using whole genome-based cross-species and intra-species analysis, we provide evidence for the unique functional specialization of Chromosome 4 through extensive acquisition of novel genes. In contrast to other insect genomes, in the sleeping chironomid a uniquely high degree of subfunctionalization in paralogous anhydrobiosis genes occurs in this chromosome, as well as pseudogenization in a highly duplicated gene family. Our findings suggest that the Chromosome 4 in *Polypedilum* is a site of high genetic turnover, allowing it to act as a ‘sandbox’ for evolutionary experiments, thus facilitating the rapid adaptation of midges to harsh environments.

## INTRODUCTION

Chironomids (non-biting midges) are one of the most widely distributed groups of insects and include cosmopolitan species and others living in niche adverse environments, where they are subjected to extremes of pH, salinity, or temperature, as well as desiccation (1,2). As these adaptation capabilities are lineage specific rather than conserved widely throughout chironomids, the extent of this rapid acquisition of novel tolerance capabilities suggests that there may be a fundamental mechanism that supports such evolution.

One of the most striking examples of adaptation of chironomids to extreme environment is the ‘sleeping chironomid’ *Polypedilum vanderplanki* (3). Upon desiccation, the larvae enter an ametabolic reversible dry state—anhydrobiosis. Loss of water causes devastating damage to living cells, and therefore anhydrobiotes have acquired various protective mechanisms to withstand such damage (4–16). We have previously showed that anhydrobiosis is mediated by evolution of unique protective gene groups highly specific for this insect, tandemly duplicated forming multiple gene clusters within the genome termed Anhydrobiosis Related Islands (ARIds) (11). These regions contain genes encoding intrinsically disordered protein LEAs, antioxidant GSTs, globins, etc., many of which have been implicated to anhydrobiosis. In comparison, the genome of the non-anhydrobiotic relative *P. nubifer* has no similar regions, thus suggesting that the genomic evolutions underlying anhydrobiosis acquisition is extremely rapid (∼50MYA). Together, we hypothesized the existence of a genomic feature that enabled such adaptations.

Genomic evolution in Diptera has been extensively studied using the *Drosophila* complex (17,18). Additional resources has also implied genome variance through expansion/contraction of genome size and constant karyotypes (19). Chironomids in general have smaller genome size (100–200 Mb) compared to the average of dipterans and insects (19,20). On the contrary, most chironomid species have additional chromosomes (chironomids: 2*n* = 6–16, mosquitoes: 2*n* = 6, flies : 2*n* = 6–8) (21,22). Several lineages even show large diversity in chromosome numbers within a single genus (22). These observations may imply a potential mechanism where chironomids genomes may have evolved through chromosomal rearrangements rather than genome enlargement and have utilized these additional genome resources for the environmental adaptations discussed above.

*P. vanderplanki* has four chromosomes (2*n* = 8) (23), of which Chromosome 4 has the highest karyotypic diversity and genetic distance in comparison with the recently described anhydrobiotic close relative *Polypedilum pembai* (23–25). This chromosome differs from the ‘dot chromosome’ found in *Drosophila*, as they are highly repetitive, heterochromatic and harbors very few genes. Chromosome 4 is half in length of the other three chromosomes and has multiple active transcriptional regions (Balbiani rings), thus would consist of much more active genes (23). These observations imply Chromosome 4 as a starting point to determine the basis of rapid genomic evolution that enabled anhydrobiosis in *P. vanderplanki*. A key factor that would be a candidate for such genomic adaptations would be the ARId loci; how are these loci spaced within the chromosome structures? What facilitated the emergence of such loci? The answers to these questions may address genome adaptations against extreme environmental stress in not only *Polypedilum*, but in chironomids, possibly insects as well. A chromosomal level genome assembly would be required to conduct such comprehensive inter-chromosome analysis; however, our previous genome assembly of *P. vanderplanki* was fragmented, hindering such analysis.

To this end, by using a combination of long-read sequencing technologies and Hi-C scaffolding, we assembled a chromosome-level genome of *P. vanderplanki*. We also applied Cap-trap RNA-Seq (CTR-Seq) to concurrently identify gene borders and promoter regions (26), which would provide rich information to following studies aimed to validate anhydrobiosis machinery identified by genomic analysis. We found that anhydrobiosis-associated genes are localized preferably on Chromosome 4, including seven out of the nine ARId loci. Using comprehensive inter-species analysis, we confirmed that most of the genes localized in this chromosome are highly specific for *P. vanderplanki*, and do not have orthologs in other Diptera. Combined with the evidence of pseudogenization in a highly duplicated gene family and unexpectedly high mutation load compared to other chromosomes, our findings suggest that the Chromosome 4 in chironomids is a unique site of high genetic turnover, allowing it to act as a ‘sandbox’ for evolutionary experiments, thus facilitating the rapid adaptation of midges to harsh environments.

## MATERIALS AND METHODS

Details can be found in SI Appendix.

### Specimen and cell culture

*P. vanderplanki* larvae were reared on 1% agar containing 2% commercial milk under controlled conditions (13 h light: 11 h dark, 27ºC) according to the previously described protocol (4). The strain used in this study was inbred for at least four generations (inbreeding code 4aG21b) and was registered as *P. vanderplanki* NIAS01. Larvae used in the experiments were starved for 24 h prior to DNA or RNA extraction. Pv11 cells were cultured in IPL-41 medium (Thermo Fisher Scientific, Waltham, MA) supplemented with 2.6 g/L Bacto™ Tryptose Phosphate Broth (Becton, Dickinson and Company, Franklin Lakes, NJ), 10% (v/v) fetal bovine serum (US origin; MP Biomedicals, Santa Ana, CA) and 0.05% (v/v) of an antibiotic and antimycotic mixture (penicillin, amphotericin B and streptomycin; Merck KGaA, Darmstadt, Germany). Cell passage was conducted every seven days.

### Genome and transcriptome sequencing

Pv11 cells were submitted to genomic DNA extraction for Illumina and PacBio long read sequencing. A library for PacBio RS II (Pacific Biosciences, Menlo Park, CA) sequencing was constructed with the 20-kb Template Preparation Using BluePippin™ Size-Selection System (Sage Science, Beverly, MA). Four libraries was constructed for Illumina sequencing; 180 and 500 bp insert libraries was constructed from the DNA extracted for PacBio sequencing with TruSeq Nano DNA Library Prep Kit (Illumina, San Diego, CA) and sequenced with HiSeq 2000 platform (Illumina), two more libraries (400–550 bp fragments) was constructed with NEBNext Ultra DNA Library Prep Kit (New England Biolabs, Ipswich, MA) and were sequenced with the MiSeq sequencer (Illumina, 262 bp paired end) or HiSeq 2500 Sequencer (100 bp paired end). Mate pair libraries (397 bp insert) was constructed with TruSeq DNA Sample Preparation kit v.2 and sequenced with HiSeq 2000 sequencer (Illumina, 100 bp paired end). Hi-C libraries were processed according to the general protocol as described previously (27) and library constructed with NEBNext Ultra II DNA Library Prep Kit for Illumina (New England Biolabs) and sequenced on a HiSeq 2500 system (Illumina, 100 bp, paired end). Library lengths were assayed with Agilent 2100 Bioanalyzer (Agilent Technologies, Santa Clara, CA). In addition, *P. vanderplanki* larvae and Pv11 cells were exposed to a variety of stress inducers (larvae: UVC 100 mJ/cm, Pv11 cells: 20.5% (v/w) NaCl, 600 mM mannitol, 600 mM trehalose, 20 μM paraquat and 42°C heat shock, anhydrobiosis cycle). Exposed larvae/cells were sampled at designated time points and total RNA was extracted with RNAiso Plus (Takara Bio, Kyoto, Japan). The sequencing library was constructed with TruSeq RNA Sample Prep Kit -v8 and was submitted to sequencing with HiSeq1500. Finally, total RNA for insect and Pv11 undergoing anhydrobiosis were also submitted to CTR-Seq library construction following a previous study (26).

### Genome assembly and gene prediction

We employed a meta-assembly to obtain a highly contiguous and complete genome assembly. Long read based genome assemblies were produced with HGAP4 and DBG2OLC (28–30), which were used for meta-assembly of an Illumina-based Platanus assembly (31). This assembly was scaffolded with Hi-C reads, resulting in the final assembly. The initial gene prediction was produced with the Braker pipeline using all RNA-Seq data stated above (32). Furthermore, Nanopore-cDNA-Seq data produced by CTR-Seq was used to predict the final Pv_5.2.4 gene set using Talon (33). This gene set was validated for completeness with BUSCO (34). The genome was submitted to variant calling with the GATK pipeline using our previous DNA-Seq data of wild *P. vanderplanki* populations, inbred NIAS01 and Pv11 (11,24,35), and Sniffles for PacBio data (36). Statistics for variants comparison were calculated with PoPoolation2, snpEff and bcftools (37–39). General features of the genome (i.e. GC ratio, Gene and repeat content, DNA-Seq and RNA-Seq coverage) were calculated in 50 or 100 kb windows with BEDtools v2.29.2–39-g5210e6f (40) or in-house Perl scripts using G-language GAE (41).

### Comparative genomics

Dipteran genomes and transcriptome assemblies were obtained from ENSEMBL Metazoa, NCBI and other data servers. For genomes without gene predictions, the Augustus gene model created during a BUSCO run was used for ab initio gene prediction (42). Transcriptome assemblies with low BUSCO scores were submitted to re-assembly with Trinity (43) to obtain higher quality transcriptome assemblies. The longest isoforms for each gene were submitted to OrthoFinder clustering (44). The amino acid sequences for each genome were pooled and bidirectional hits were determined with Diamond blastp v0.9.24 (45) and the number of genes conserved between species were calculated with in-house Perl scripts. In addition, 1-to-1 ortholog were determined for *Drosophila melanogaster* to identify *D. melanogaster* essential gene orthologs. Each chromosome was tested for enrichment or depletion of Dm-essential gene orthologs or BUSCO genes. Colinear blocks were detected with McScanX and visualized with synvisio (46,47). dN/dS ratio between *P. vanderplanki* and *P. pembai* orthologs were calculated with codeml in the PAML package (48) based on MAFFT alignments (49,50). Gene ontology enrichment analysis for several gene groups were performed with GOStats v2.52.0 (51).

### Transcriptome analysis

Gene expression was quantified using RSEM in the Trinity package and were submitted to differential expression analysis with DESeq2 and edgeR (52–54). Genes with FDR < 0.05 and FC > 2 was designated as differentially expressed genes. Gene expression profiles were clustered based on the Spearman correlation, and genes in each cluster were submitted to gene ontology enrichment analysis using GOStat and visualized with Revigo (51,55).

## RESULTS

### A chromosomal-scale genome assembly for *Polypedilum vanderplanki*

**Figure 1.**
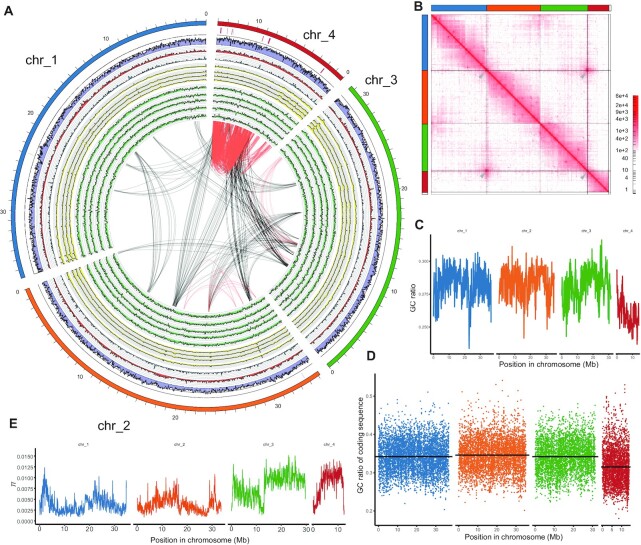
Chromosome-scale genome assembly of *Polypedilum vanderplanki*. (**A**) Circos plot of the four chromosomes. From the outer rim, ARId regions, AT% (purple), gene count for each 50 Kbp window (red), non-coding gene count for each 50 kb window (blue), genome coverage of Illumina DNA-Seq (SRR12736661, SRR12736660, SRR12736662, SRR12736659, yellow), RNA-Seq coverage (DRR024752, DRR024753, DRR024754, DRR024755, DRR024756, green), collinear blocks calculated with MCScanX (black: inter-chromosomal, red: intra-chromosomal). Colors used for each chromosome are inherited in subsequent figures. Individual figures are indicated on Additional Figure S1 (**B**) Contact map of Hi-C reads. Hi-C reads were mapped to the genome assembly and KR transformed contact frequencies at 250kbp were visualized as a contact map with Jucier. Gray arrowheads indicate possible telomeric regions. (**C**) GC ratio of 100 kb windows. (**D**) GC ratio of the coding sequence from the longest isoform for each gene. The black lines indicate the chromosome average (chr_1: 34.20%, chr_2: 34.60%, chr_3: 34.17%, chr_4: 31.50%). (**E**) Pairwise nucleotide diversity (*π*) of 50 kb windows. An increase in *π* diversity can be observed in the latter half of Chromosome 3 and most of Chromosome 4.

We assembled a chromosome-level genome for *P. vanderplanki*—the first chromosome-level genome in any anhydrobiotic animals—utilizing Illumina, PacBio and Hi-C sequencing data from *P. vanderplanki*-derived Pv11 cell line and larvae (Additional Data S1, S2 and S3, Additional Text S1). The 98.8% of the resulting 118.9 Mb assembly is arranged in the four largest scaffolds (Table 1), corresponding to its four chromosomes (Figure 1A). High BUSCO scores were observed (Diptera lineage, Complete 95.7%, Missing 3.6%, Additional Text S1) and repeat sequences detection combined with Hi-C contact mapping suggested the existence of putative centromeric and telomeric regions, respectively (Figure 1B, Additional Data S4, Additional Text S1). Comparison of Pv5.2 with our previous genome assembly Pv0.9 did not show extensive genomic changes that would be anticipated from sequencing the Pv11 cell line (Additional Text S2, Additional Figure S2).

**Table 1.** Statistics of *P. vanderplanki* genome

	Pv0.9	Pv5.2
Main genome scaffold total	9104	388
Main genome contig total	25 913	2067
Main genome scaffold sequence total (Mb)	116.771	118.969
Main genome contig sequence total (Mb)	111.894	118.352
% Gap	4.177	0.518
Main genome scaffold N/L50	101/264.32 kb	2/35.209 Mb
Main genome contig N/L50	2191/12.463 kb	161/219.078 kb
Main genome scaffold N/L90	1986/4.421 kb	4/14.02 Mb
Main genome contig N/L90	10 658/2.139 kb	596/49.999 kb
Max scaffold length	2.184 Mb	36.877 Mb
Max contig length	358 kb	1.494 Mb
Number of scaffolds >50KB	358	7
% Main genome in scaffolds >50KB	77.60	98.98
% GC	28.3%	28.1%
% N	4.2%	0.5%
BUSCO4 completeness (Diptera)	92.9% [91.9%,1.0%], 2.7%, 4.4%	95.7% [94.7%,1.0%], 0.6%, 3.7%
BUSCO4 completeness (Insecta)	96.4% [95.4%,1.0%], 1.5%, 2.1%	98.3% [90.9%,7.4%], 0.5%, 1.2%

We combined all available RNA-Seq data and full-length cDNA Nanopore sequencing from Cap Trap RNA-Seq (CTR-Seq) (26) to concurrently identify regulatory regions, full-length transcripts, and previously unidentified long noncoding genes (Table 2, Additional Figure S3, Additional Data S5 and S6, Additional Text S3 and S4). This resulted in the Pv5.2.4 gene set composed of 18 989 genes (65 981 transcripts) with a BUSCO4 completeness score of 96.2% (Diptera lineage), in line with other dipterans. We detected 0.39–0.47% of horizontally transferred genes, consistent with values determined in Diptera (56) (Additional Text S4). There was no significant enrichment of horizontally transferred genes in any specific chromosomes (Additional Text S4).

**Table 2.** Statistics of gene structure obtained by TALON

Data preparation						
Category	Genome		Braker		Final	
Version	Pv_5.0		Pv_5.0.1		Pv_5.2.4	
**Gene numbers**						
**Category**	**#**	**Percent**	**#**	**Percent**	**#**	**Percent**
Total genes	-	-	17 852	100.00%	18 990	100.00%
Total transcripts	-	-	19 117	107.09%	67 079	353.23%
**BUSCO completeness**						
	**Insecta**	**Diptera**	**Insecta**	**Diptera**	**Insecta**	**Diptera**
Complete	98.3	95.7	98.3	95.9	98.6	96.2
Single	97.1	94.7	90.9	55.3	50.3	57.0
Duplicated	1.2	1.0	7.4	40.6	48.3	39.2
Fragmented	0.2	0.6	0.5	0.9	0.4	1.0
Missing	1.5	3.7	1.2	3.2	1.0	2.8
**Mapping ratio to transcript sequences (bwa mem)**						
**CTR-Seq mRNA-Seq reads**	**#Reads**	**Percent**	**#Reads**	**Percent**	**#Reads**	**Percent**
Wet-1	-		95 103 034	84.24%	104 346 021	92.19%
Wet-2	-		102 208 115	84.32%	111 736 595	91.96%
D24-1	-		106 902 973	83.57%	119 406 151	93.12%
D24-2	-		101 771 203	82.68%	114 237 766	92.55%
D48	-		113 995 064	83.84%	127 677 786	93.61%
Pv11-T0	-		98 544 418	80.09%	111 471 188	90.23%
Pv11-T48	-		91 836 757	79.69%	103 011 915	89.12%

These statistics indicate that this genome assembly has greatly improved contiguity, completeness and quality compared to our previous genome resources, and is compatible for comparison with other Diptera genomes.

### General analysis revealed unusual features of Chromosome 4

Upon gene model annotation and general analysis, we noticed that there are unexpected, but clear inequalities among chromosomes in terms of several key characteristics. The majority of previously identified anhydrobiosis genes (i.e. *Lea* and *Lil* genes) are located on Chromosome 4; seven out of the nine ARIds were located on Chromosome 4 (Figure 1A, Additional Data S7). Intra-chromosomal synteny regions were increased on Chromosome 4 than other chromosomes (Figure 1A). Several transposable elements (TEs) in were enriched in Chromosome 4 (Additional Data S8). Chromosome 4 has lower average chromosome-wide GC ratio (Figure 1C) and at gene level (Figure 1D, Tukey HSD, FDR < 0.001). These features implied Chromosome 4 may harbor other features that may have provided the basis of genomic evolution in *P. vanderplanki*.

Hypothesizing that this decrease in GC nucleotides may be related to mutation accumulation, we profiled the genome for variants with our previous Pool-Seq data of wild *P. vanderplanki* populations (Additional Data S9, Additional Figure S4). We observed increased pairwise nucleotide diversity in the 3–12 Mb region of Chromosome 4 (Figure 1E, Additional text S5), implying relaxed selective pressure in these regions. On the contrary, lower Ts/Tv ratio in Chromosome 4 were observed (Additional Data S9, Additional Figure S4d, Tukey HSD, FDR < 0.01), suggesting a biased substitution profile compared to other chromosomes.

Together, Chromosome 4 has a highly biased nucleotide composition and accumulation of variants compared to other chromosomes, suggesting an increase in the genetic diversity of Chromosome 4, possibly caused by relaxed selective pressure. Thus we hypothesized that Chromosome 4 may specifically harbor traces of genomic adaptations that enabled anhydrobiosis in this species.

### Chromosome 4 lack synteny blocks conserved in other Diptera but is not of completely novel origin

We then proceeded to compare the genomic content of *P. vanderplanki* with that of publicly available dipterans and chironomids to identify genetic content specific to *P. vanderplanki*, and more interestingly, Chromosome 4 (Additional Data S10, Additional Text S6 and S7).

**Figure 2.**
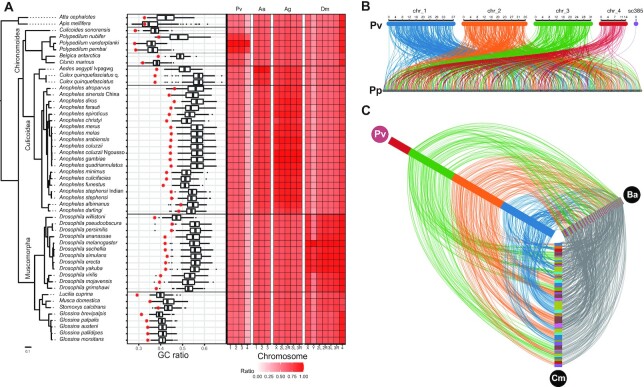
Chromosome 4 lacks synteny blocks with other Diptera. (**A**) GC ratios and gene conservation ratios between Dipteran species. Conservation ratio and GC ratio (genome and single copy genes) were plotted against a phylogenetic tree using 1,014 single copy orthologs from Orthofinder clustering of longest isoforms. Homologs were determined by reciprocal diamond blastp searches and conservation ratios were counted for each chromosome of *P. vanderplanki*, *A. aegypti*, *A. gambiae* and *D. melanogaster*. A red asterisk indicates genome-level GC ratio. Only autosome and sex chromosomes are visualized (unplaced scaffolds are skipped). (**B**, **C**) Detection of collinear blocks between [b] the two *Polypedilum* species (*P. vanderplanki* and *P. pembai*) and (C) the family Chironomidae (*B. antarctica*, *C. marinus* and *P. vanderplanki*). Amino acid sequences of longest isoforms were submitted for diamond blastp searches and collinear blocks were detected and visualized with McScanX and synvisio. Links are colored according to the chromosome color used in Figure 1A.

As we have hypothesized that the decrease in GC ratio may be related to the genomic evolution occurring in *P. vanderplanki*, we first assayed the GC ratio within Diptera. We observed low GC ratio in multiple chironomids compared to other Diptera (Figure 2A). Genomes with lower GC ratio showed lower GC ratio at the coding sequences level as well, suggesting a genome-wide adaptation. Interestingly, the desiccation-sensitive *P. nubifer* and the Antarctic midge *Belgica antarctica* did not show such reductions (11,57), inferring a dynamic accumulation of G/C nucleotides after their divergence with other lineages.

We then analyzed the gene conservation ratio, e.g. number of genes in a chromosome that a homolog could be detected in other dipterans. Genes on Chromosome 4 (3 241 genes) showed lower conservation (30–40%) with chironomids outside of the subfamily Chironominae, compared to those on other chromosomes (55–70%, Figure 2A). Using other major dipteran chromosome-level genomes (*Aedes aegypti*, *Anopheles gambiae*, *D. melanogaster*) did not show such decreases in conservation ratios in a single autosome, with the exception of the Y chromosome in *D. melanogaster*. Using draft genome and transcriptome assemblies of chironomids (Additional Text S6, Additional Data S11) also indicated similar conservation profiles (Additional Figure S5a, Additional Text S7). Besides, by using *ab initio* gene predictions for all genomes, we verified that using different gene prediction methods did not affect our results (Additional Text S7, Additional Figure S5b). These data suggests that Chromosome 4 may harbor a large number of species (or genus) specific genes.

These data led us to consider whether Chromosome 4 genes were evolutionary conserved from a common Diptera ancestor. Thus, we assayed whether synteny blocks would be detected between *P. vanderplanki* and other dipterans. Collinear blocks were detected between *P. vanderplanki* and *P. pembai* in all chromosomes (Figure 2B), but all other species studied lacked such blocks on Chromosome 4 (Culicomorpha and Chironomidae, Figure 2C, Additional Figure S5c). Results varied in close relatives (Chironomini tribe, e.g. *P. nubifer*, *Chironomus tentans*, *Chironomus riparius*, Additional Figure S3def). The lack of synteny blocks between *P. vanderplanki* and other chironomids may be due to the fragmentation of the genome assembly (i.e. *P. nubifer* has 9672 scaffolds, N50 length 26kbp), preventing correct synteny analysis. Assemblies with higher contiguity would be required for more comprehensive analysis.

This observation led us to question where the genes on Chromosome 4 genes originated from. By determining orthologs by bidirectional best hits, we observed that the genes on Chromosome 4 have orthologs across all chromosomes in *D. melanogaster*, *A. aegypti*, and *A. gambiae* (Additional Figure 5g, Additional Text S7), indicating that this chromosome was not obtained through horizontal transfer. This also suggested that inter-chromosomal translocations have occurred at some time point during the evolutionary trajectory of chironomids, creating a mosaic conservation pattern. Intriguingly, we observed that Chromosome 4 was significantly depleted of BUSCO genes (Diptera lineage) or *Drosophila* essential genes (Additional Data S13). Furthermore, higher dN/dS values were observed between 1-to-1 *P. vanderplanki* and *P. pembai* Chromosome 4 orthologs (Additional Figure S5h), suggesting relaxed selective pressure on Chromosome 4 genes.

Together, these observations suggest that Chromosome 4 is not of novel origin and shares genes with all chromosomes in other Mosquitoes. While we observed relaxed selection on Chromosome 4 genes, we also found contradicting data suggesting the existence of a selective pressure to remove essential or highly conserved genes from Chromosome 4.

### Novel genes on Chromosome 4 are enriched in ionotropic receptor pseudogenes

**Figure 3.**
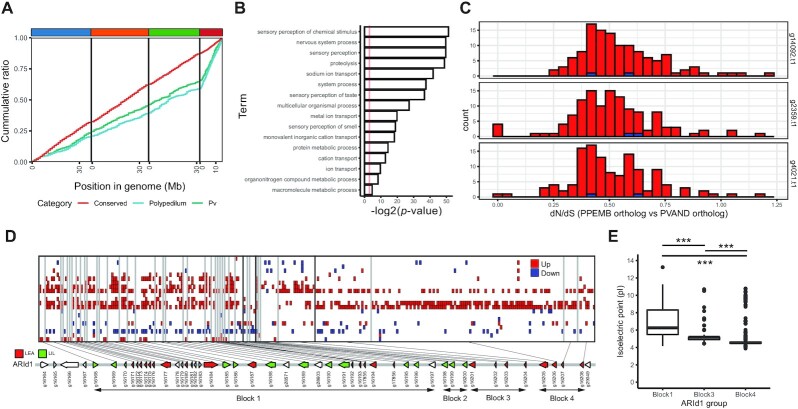
Functional and non-functional multi-copy ortholog groups. (**A**) The cumulative number of genes specific to *P. vanderplanki*, specific to the genus *Polypedilum*, and conserved within Diptera (Conserved), along the genome. Clade specificity was determined by gene counts of OrthoFinder ortholog groups. (**B**) Gene ontology enrichment analysis of *Polypedilum*-specific genes on Chromosome 4. Only terms in the Biological Process category are shown. (**C**) dN/dS values between the three *P. pembai* orthologs (g14092.t1, g2359.t1, g4021.t1) and *P. vanderplanki* orthologs. Coding sequences were aligned with MAFFT, and dN/dS values were calculated with codeml. (**D**) Differential expression information of LEA protein gene orthologs in ARId1. Conditions identified as differentially expressed (from top row : (1) Heat 42°C T1; (2) Heat 42°C T24, (3) Paraquat T1; (4) Paraquat T24, (5) Mannitol T3; (6) Mannitol T24; (7) NaCl T1; (8) NaCl T3; (9) NaCl T24; (10) Trehalose T1; (11) Trehalose T3; (12) PreCondTre T0vsT12; (13) PreCondTre T12vsT24; (14) PreCondTre T24vsT36; (15) PreCondTre T36vsT48; (16) PreCondTre T48 versus Rehydration T0; (17) Rehydration T0 versusT3; (18) Rehydration T3vsT12; (19) Rehydration T12vsT24; (20) Rehydration T24vsT72) are indicated in colors (up-regulated : red, down-regulated : blue). (**E**) The pI values of proteins deduced from *Lea* orthologs in each Block. *** *p*-value < 0.001

We then turned to novel genes on Chromosome 4; are there any functions that are enriched in genes located on this highly variable chromosome? Approximately 40% of the *P. vanderplanki*- or *Polypedilum*-specific genes mapped to Chromosome 4 (Figure 3A, Additional Data S14, Additional Text S8), suggesting that this chromosome might function as a scaffold for acquisition of novel genes. We found 327 ortholog clusters, corresponding to 653 genes, with orthologs in all *Polypedilum* species but not in other dipterans or outgroups, of which 137 genes had a Swiss-Prot homolog (BLASTP, 1e–15). Gene ontology enrichment analysis of these genes based on InterPro annotations indicated enrichment of chemosensory functions and ion transportation (Figure 3B, Additional Data S15). We also observed that Chromosome 4 harbored genes that were not expressed in any of the transcriptome samples sequenced in this study—at nearly twice the rate of other chromosomes (Table 3)—suggesting a higher number of pseudogenes on Chromosome 4.

**Table 3.** High proportion of non-expressed genes on Chromosome 4

	Chromosome 1		Chromosome 2		Chromosome 3		Chromosome 4	
Length (bp)	36 877 143	31%	35 209 052	30%	31 432 203	27%	14 019 908	12%
Genes								
Total	5275	30%	4911	28%	4197	24%	3241	18%
No. gene/Mb	143.0		139.5		133.5		231.2	
Protein coding	4908	93%	4588	93%	3918	93%	3136	97%
Non-coding	367	7%	323	7%	279	7%	105	3%
Gene TPM > 1	2987	57%	2738	56%	2215	53%	1156	36%
Gene TPM ≦ 1	2288	43%	2173	44%	1982	47%	2085	64%
TPM > 1/Mb	81.0		77.8		70.5		82.5	

We then filtered the ortholog clusters (Clustering 1, Additional Text S8) for *Polypedilum*-specific gene families. Intriguingly, eight out of the largest 15 clusters were transmembrane proteins, including the LIL protein family. Proteins in four clusters were annotated with the ionotropic receptor domain 11a or 20A, but BLASTP search against Swiss-Prot did not show homology to previously known ionotropic receptors (*E*-value < 1e–15 threshold). The largest ortholog cluster (OG0000168) was extensively duplicated in *P. vanderplanki* (117 copies, *P. pembai* three copies, *P. nubifer* one copy, Additional Data S16) with 45 copies on Chromosome 4 (significantly enriched, Fisher's exact test, *p*-value = 1.38 × 10^–7^). Most orthologs (115/117 copies in OG0000168), were not expressed (average TPM < 1); we hypothesize that these are pseudogenes. High number copies of non-expressed genes also implied transposon activity; however, only one gene was predicted to have a Dfam domain (DR0206503, *E*-value = 8.5e–05), thus showing this protein family was not obtained from transposon activity. Calculating dN/dS values between all *P. pembai* and *P. vanderplank*i orthologs indicated an average of 0.51–0.55 (median 0.48–0.52, Figure 3C), considerably higher than dN/dS values of *P. pembai* and *P. vanderplanki* 1-to-1 orthologs (Additional Figure 5h) or gene families of similar size (24). These data support that this gene family may be under relaxed selection, due to pseudogenization or its multicopy nature.

### Subfunctionalization of *Lea* orthologs on Chromosome 4 support anhydrobiosis

Finally, we concentrated on anhydrobiosis genes on Chromosome 4. All ARId loci were present in the genome, with seven out of the nine regions being placed on Chromosome 4 (Additional Data S7). Most of these regions are located at the latter half of Chromosome 4, which has higher nucleotide diversity and lower GC ratio. This observation emphasized the importance of the features of Chromosome 4 indicated above, as it may possibly shape the basis of the rapid environmental adaptation in various lineages.

ARIds are loci enriched in genes upregulated during anhydrobiosis entry. For example, the largest region, ARId 1, harbors 22 LEA and 14 LIL protein coding genes. Previous studies have identified paralog specific cellular localization, protein characteristics, and gene expression profiles of these paralogs (12,16). We extended this analysis by conducting transcriptome analysis of Pv11 cells subjected to various stresses (Additional Figure S6, Additional Data S17 and S18) and identified differentially expressed transcripts with stringent thresholds. Specific discussion on the analysis of transcriptome profiles can be found in the Additional text S9.

As previous studies have suggested, various genes in the ARId regions were differentially expressed, supporting our previous findings. We focused on the ARId1 locus, as it harbors the most anhydrobiosis related genes duplicated in tandem and on the same strand. Phylogenetic analysis has indicated many of the closely located *Lea* paralogs originate from a single ancestor, making it an ideal circumstance for generating subfunctionalization between paralogs. The Pv5.2.4 gene set contains 2 178 transcripts originating from ARId1 (corresponding to 50 genes). We detected 496 transcripts with an average TPM over 1, of which approximately 281 transcripts (42 genes) were differentially expressed in our time course transcriptome data. Grouping consecutive genes by their regulation profiles (e.g. regulated during either Mannitol/NaCl exposure or Trehalose preconditioning, or both) allowed us to divide them into four ‘Blocks’ (Figure 3D, Additional Text S10). We observed that the Blocks expressed widely across datasets had significantly higher isoelectric point (pI) values (average 6.87) of deduced proteins than the two other *Lea* Blocks (average 5.18 and 4.74; Tukey HSD, Figure 3E). The regulation groups were characterized by the presence of heat shock elements (HSEs): similarly, four out of five orthologs in Block 4 had HSEs in their upstream sequences. We have previously shown the importance of gene regulation by Heat Shock Factor (HSF) in Pv11 cells, thus identification of HSEs in Block 4 *Lea* paralogs would support their contribution to anhydrobiosis. These findings suggests that paralogous anhydrobiosis loci, at least *Lea* genes in this case, may have undergone subfunctionalization to accommodate dynamic changes in cellular physiology that may occur during anhydrobiosis.

## DISCUSSION

This study illustrates a single chromosome playing an important role in the rapid adaptation in *P. vanderplanki*, providing evidence of chromosome-specific mechanisms of evolution in insects in general. It is known that basal chironomid species have more diverse numbers of chromosomes (2*n* = 6–16) compared to other insects (22). Systematic karyotypic studies of polytene chromosomes in chironomid midges have shown that a change in the basic chromosomal band sequence in arms A, B, C, D, E, F (representing Chromosomes 1-3) happens only when the basic chromosomal band sequence in arm G (Chromosome 4) also changes (58). Specifically, for chironomid midges, the fourth chromosome at the micromorphology level show the highest variation among populations exposed to different stresses (59). The high-order structure of the chromosomes may have facilitated this chromosome-level adaptations. Heterochromatin (HC) is prone to fuse with itself at low temperatures, leading to merging of chromosomes at chromocenters, tandem chromosomal merging, reciprocal exchange of chromosomal arms and the creation of new cytocomplexes. HC has a nonspecific reaction to environmental stresses, such as unusual salinity or temperature, which results in condensation-decondensation of HC at the nuclear membrane. Also, the presence of HC provides a high level of chromosomal reconstruction and a change in HC content in different species could have adaptive value (60).We have yet to validate genome-wide heterochromatin structures; ongoing Hi-C analysis based on this chromosome level genome will be discussed elsewhere.

The analysis conducted in this study suggest that Chromosome 4 (in its 3–12 Mb region) may be under relaxed selective pressure, allowing the accumulation of heterozygotic mutations. This enabled the acquisition of novel genes as subfunctionalization after gene duplication is one route to the generation of genes with new functions. We find most of the ARIds to be in these regions, thus implicating the link between the variable Chromosome 4 and genomic adaptation against extreme stress. These observations suggest that the variable Chromosome 4 have promoted acquisition of novel gene sets that contributed to environmental adaptations. However, how adaptive variants that produced anhydrobiosis mechanisms became to be fixed on Chromosome 4 remains to be identified. Determining if the size and number of chromosomes correlates with higher diversity (61) would be another point that needs to be validated.

One element that may have caused the diversity in Chromosome 4 is DNA damage. We have previously observed extensive DNA damage during larvae anhydrobiosis and the induction of Rad51, an activator of double strand break repair during recovery (9). This implies the induction of homologous recombination repair (HR) or non-homologous end joining (NHEJ) during anhydrobiosis recovery. Our previous transcriptome analysis of Pv11 anhydrobiosis has revealed that NHEJ may be preferred in Pv11 cells (62), which if the same regulation is used in insects, would suggest some kind of regulation of HR. If this is the case, decrease of HR may have caused inhibition of GC-biased gene conversion, as it is linked with HR, thus suppressing the increase in GC ratio seen in other organisms. Furthermore, the biased substitution pattern indicated by the lower Ts/Tv values in Chromosome 4 may have been caused by the inhibition of GC-biased gene conversion. Lower Ts/Tv may also indicate oxidative stress caused during desiccation may have caused the oxidation of the guanosine nucleotide, causing G-to-T transversion, which were not correctly repaired. The relaxed selection pressure may have enhanced mutation accumulation caused by NHEJ repair or nucleotide-level DNA damage. Together, these mechanisms have driven the decrease in GC ratio and increase of nucleotide diversity in Chromosome 4.

Moreover, we have observed decrease in gene conservation in the Y sex chromosome of *D. melanogaster*, similar to what we observed in *P. vanderplanki* Chromosome 4. Sex chromosomes are known to have features similar to what we have observed in Chromosome 4, e.g. higher mutation rates, etc. (63). Environmental adaptation through genomic evolution on sex chromosomes in insects is rare, as we have found only one study showing thermal adaptation in the house fly *Musca domestic* linked with Y sex chromosome genes (64). The *P. vanderplanki* Chromosome 4 differs from the *D. melanogaster* sex chromosome as it contains much more genes and is not highly repetitive. Chironomids lack definitive sex chromosomes, queried through synteny analysis with Müller elements from *Drosophila* (65); however, sex determining regions (SDR) and sex biased regions have been identified by molecular and karyotyping studies. In *C. riparius*, reduced recombination rate has been observed in the Chromosome 3 SDR (66). In *Polypedilum*, a previous karyotyping analysis on *P. nubifer* has identified a sex-biased region on Chromosome 4 (67). This may suggest that the sex-biased region may be also conserved in *P. vanderplanki* Chromosome 4 and may be linked to the reduction in recombination. Furthermore, considering that the SDR is located on Chromosome 3 in *C. riparius*, establishment of Chromosome 4 as a sex chromosome may be recent. More studies based on this chromosome scale genome would provide a wide picture to the sex determination in this insect.

By comparison with an anhydrobiosis-incompetent relative, *P. nubifer*, we have previously shown that anhydrobiosis genes (*i.e*. those encoding LEA, LILs, thioredoxin proteins) have gone through massive duplication in the *P. vanderplanki* genome, forming a feature known as ARIds (11,12,16). Comparison with *P. pembai* suggested that the evolution of anhydrobiosis genes within the *Polypedilum* lineage may have occurred recently, at least after the divergence of *P. vanderplanki* and *P. nubifer* (63–100 MYA) (11,24,25,68). Most of these loci were located on Chromosome 4, emphasizing the importance of this chromosome for adaptation against desiccation.

Close examination of genomic loci on Chromosome 4 revealed two examples of genomic evolution on this chromosome. Chromosome 4 harbors majority of the *Polypedilum* or *P. vanderplanki* specific genes. From functional enrichment analysis, we identified a high copy number pseudogene family with weak homology with ionotropic receptors. Ionotropic receptor domains are thought to participate in chemical stimulus sensing (69). Chemosensory genes have been proposed to undergo subfunctionalization through duplication, facilitating the development of novel ecological traits (70). We observed relaxed selection in the orthologs, together with their expression profiles support these orthologs being pseudogenes. This data proposes that these genes may be remnants of gene duplication events that occurred after *P. vanderplanki and P. pembai* diverged approximately 33 MYA. It is possible that this protein family functioned as a desiccation sensor in the early stages of acquisition of the anhydrobiosis machinery, but eventually became unnecessary.

Similar to the accumulation of genus specific genes, Chromosome 4 harbors most of the ARId loci. In particular, ARId1 harbors the massively duplicated *Lea* and *Lil* genes. Previous studies have proposed that duplication of LEA protein genes allows their subfunctionalization to improve adaptation to desiccation (12,71). We have observed enrichment of HSEs in the upstream regions of Block 4 *Lea* paralogs and significantly higher pI values of Block 1 paralogs. These observations suggest the subfunctionalization in these paralogs; LEA proteins with pI values close to neutral are expressed when cells face osmotic stress, whereas paralogs with lower pI values are produced under other conditions. Intrinsically disordered proteins, in particular LEAs, have been implicated in various cellular mechanisms, including phase transition and protection of cellular molecules in anhydrobiotes (72–76). A previous study using measles virus phosphoprotein showed that such proteins are less soluble at their isoelectric points (77). If this also is the case for *P. vanderplanki* LEA proteins, it may suggest that duplications of LEA protein genes have occurred to enable cellular protection when facing drastic changes in cellular pH, such as those that may occur during trehalose preconditioning and subsequent dehydration in Pv11 cells or desiccation in larvae. This subfunctionalization may have been facilitated by the relaxed selection taking place on Chromosome 4; the loci harboring multiple *Pimt* paralogs (ARId3) also shows similar signs (24). Moreover, the existence of HSEs in Block 4 *Lea* paralogs expands our knowledge on these ARId regions; we have previously identified HSF to be a major regulator of anhydrobiosis genes (78). Knock out experiments using the CRISPR/Cas9 system in Pv11 cells causes defects preventing cells from successfully entering anhydrobiosis (79). We anticipate examination of other ARId loci will present similar features linking currently identified transcription factors with effective proteins.

In conclusion, we present a chromosome-level genome assembly of the anhydrobiotic midge *P. vanderplanki*, and have observed for the first time, to our knowledge, the use of a single chromosome for trial-and-error genetic modifications leading to rapid environmental adaptation. The evolution of *P. vanderplanki* Chromosome 4 may have been fundamental to the rapid evolution of novel chironomid physiologies, i.e. tolerance to desiccation, by accumulation of novel genes. The relaxed selective pressure on Chromosome 4 has likely contributed to the rapidity of evolution. We provide two examples of such evolutionary experiments: subfunctionalization of the ARId1 *Lea* loci that underpin anhydrobiosis, and pseudogenization of a 117-copy ionotropic receptor gene family, possibly illustrating the evolutionary trajectory of anhydrobiosis in *P. vanderplanki*. Acquisition of such adaptive mechanisms may have enabled *P. vanderplanki* to inhabit environments where there is less competition. Together, this study demonstrates the genetic basis of desiccation tolerance within the *Polypedilum* genus and infers a similar mechanism for the adaptation to extreme environments in other chironomids. We await more chromosome-level chironomid genome sequences to verify the universality of additional chromosomes as a genetically plastic ‘sandbox’ in which to test evolutionary strategies for environmental adaptation in chironomids.

## DATA AVAILABILITY

All sequencing data and the assembled genome obtained in this study has been uploaded to NCBI. A Jbrowse-based genome browser for the *P. vanderplanki* genome is hosted at MidgeBase2 (https://www.midgebase.org). Raw data and scripts used in this study were uploaded to a dedicated GitHub repository (https://github.com/Kikawada-Lab-UT-NARO/Pvanderplanki_chromosomal_genome).

The genome, DNA and CTR-Seq sequencing data and annotations have been uploaded to NCBI under the accession ID PRJNA660906. The transcriptome sequencing data have been uploaded to GEO under the accession ID GSE158443. This Whole Genome Shotgun project of *P. vanderplanki* implemented with the Braker gene prediction (v5.2) has been deposited at DDBJ/ENA/GenBank under the accession JADBJN000000000. The version described in this paper is version JADBJN010000000.

## Supplementary Material

lqac029_Supplemental_Files

## Notes

Present address: Yuki Yoshida, Department of Multidisciplinary Sciences, Graduate School of Arts and Sciences, The University of Tokyo, Meguro, Tokyo 153-0041, Japan.
