# Supplementary material for: High quality genome assembly of the anhydrobiotic midge provides insights on a single chromosome-based emergence of extreme desiccation tolerance
Source: NAR Genom Bioinform. 2022 Apr 5;4(2):lqac029. doi: 10.1093/nargab/lqac029 (PMC8982440; doi:10.1093/nargab/lqac029)
Supplement: lqac029_Supplemental_Files [file lqac029_supplemental_files.zip › NAR_SI_final.pdf]

Additional Information for

High quality genome assembly of the anhydrobiotic midge provides insights on a single chromosome-based emergence of extreme desiccation tolerance

Yuki Yoshida, Nurislam Shaikhutdinov, Olga Kozlova, Masayoshi Itoh, Michihira Tagami, Mitsuyoshi Murata, Hiromi Nishiyori-Sueki, Miki Kojima-Ishiyama, Shohei Noma, Alexander Cherkasov, Guzel Gazizova, Aigul Nasibullina, Ruslan Deviatiiarov, Elena Shagimardanova, Alina Ryabova, Katsushi Yamaguchi, Takahiro Bino, Shuji Shigenobu, Shoko Tokumoto,Yugo Miyata, Richard Cornette, Takahiro G. Yamada, Akira Funahashi, Masaru Tomita, Oleg Gusev*,Takahiro Kikawada*

This PDF file includes:

- Material and methods
- Additional text
- SI References
- Additional Figures S1 to S6
- Legends to Additional Data S1 to S18

MATERIALS AND METHODS

1. Genome and transcriptome sequencing

Pelleted Pv11 cells were homogenized with a BioMasher II (Nippi, Tokyo, Japan) in QIAGEN Buffer G2 (Qiagen, Venlo, Netherlands). Genomic DNA was extracted using QIAGEN Genomic-tip 20/G columns (Qiagen). The extracted DNA was divided into two aliquots for PacBio and Illumina sequencing. For PacBio sequencing, high-molecular-weight genomic DNA was sheared to a targeted average size of 20-30 kbp with a Covaris g-Tube (Covaris, Woburn, MA). A SMRTbell library for PacBio long-read sequencing was constructed according to the manufacturer's recommended protocol for 20 kbp inserts (the 20-kb Template Preparation Using BluePippin™ Size-Selection System, Sage Science, Beverly, MA). The SMRTbell library was sequenced on a PacBio RS II sequencer (Pacific Biosciences, Menlo Park, CA) using a DNA/Polymerase Binding Kit P6 v2 (Pacific Biosciences) and a DNA Sequencing Reagent Kit 4.0 (Pacific Biosciences). For short read Illumina sequencing, the purified DNA was fragmented with a Covaris S2 sonicator (Covaris), and DNA fragments of ~180 or ~500 bp were size selected using the Pippin Prep system (Sage Science). Two sequencing libraries were prepared using the TruSeq Nano DNA Library Prep Kit (Illumina, San Diego, CA) with insert sizes of ~180 and ~500 bp. The quality and quantity of the DNA library were evaluated by electrophoresis using an Agilent 2100 Bioanalyzer (Agilent Technologies, Santa Clara, CA) and KAPA Library Quantification Kits (Roche Molecular Diagnostics, Pleasanton, CA). These DNA libraries were sequenced on the HiSeq 2000 platform (Illumina) to generate paired-end 101-bp reads.

For preparation of the third and fourth Illumina short-read libraries, 10^7 cells were washed in PBS (Thermo Fisher Scientific) and extracted with DNeasy Blood & Tissue Kit (Qiagen). The quality and quantity of the extracted DNA were validated with a nanophotometer (Implen, Munich, Germany) and a Qubit dsDNA BR Assay Kit (Thermo Fisher Scientific). The extracted DNA was fragmented into 400-550 bp lengths with a sonicator (Qsonica, Newtown, CT). Each Illumina sequencing library was constructed with a NEBNext Ultra DNA Library Prep Kit (New England Biolabs, Ipswich, MA) according to the manufacturer's instructions and sequenced in paired-end mode 262+262 on a Miseq sequencer (Illumina) or 100+100 on a HiSeq2500 sequencer (Illumina).

To construct the mate-pair libraries, DNA was processed using a TruSeq DNA Sample Preparation kit v.2 (Illumina) according to the manufacturer's instructions. Library insert lengths, as assessed using an Agilent 2100 Bioanalyzer (Agilent Technologies), were 397 bp for *P. vanderplanki*. Libraries were quantified using fluorometry with a Qubit 2.0 instrument (Thermo Fisher Scientific) and real-time PCR and diluted to a final concentration of 9 pM. Diluted libraries were clustered using a cBot instrument (Illumina) with a TruSeq PE Cluster Kit v3 (Illumina) and sequenced using a HiSeq 2000 sequencer (Illumina) with a TruSeq SBS Kit v3-HS (Illumina), read length 101 from each end.

In situ Hi-C samples were processed according to the general protocol as described previously (1), which was adapted for use on insect cell lines. Mainly, differences related to adjustments in the volume of the reactions, speed of centrifugation and incubation timing. To fix the cells, 2×10^7 of Pv11 cells were suspended in 1 mL IPL-41 medium

and fixed for 10 min by adding formaldehyde at a final concentration of 1% at room temperature (RT) with moderate rotation. The reaction was quenched with 2.5 M glycine (added to final concentration 0.2 M) for 5 min at RT with rotation. Fixed cells were washed twice with cold 1×PBS and then lysed for 30 min at RT. Chromatin was solubilized by adding 0.5% SDS to the lysed cell pellet and incubated for 10 min at 62 °C without rotation. Chromatin digestion was performed by adding 100 U of MboI (New England Biolabs) in 250 µl NEB2 buffer per sample and incubated at 37˚C overnight with constant agitation. The overhangs of the digested DNA were labeled by adding biotin-14-dATP. The biotin-labeled DNA fragments obtained were ligated by overnight incubation at RT with gentle rotation. Crosslinks were reversed by overnight incubation with 1% SDS and proteinase K, and DNA was isolated by sodium acetate/ethanol precipitation. The samples were then sheared using a Covaris LE220 instrument (fill level – 10, duty cycle – 15, PIP – 500, cycles/burst – 200, time – 58 s). The 300-500 bp DNA fraction was isolated from samples using AMPure XP beads (Beckman Coulter, Brea, CA). Dynabeads MyOne Streptavidin T1 beads (Thermo Fisher Scientific) were used to pull down biotinylated fragments. Remaining biotin from unligated ends was removed and A-tails were added with T4 PNK. The Illumina sequencing library was prepared using reagents from the NEBNext Ultra II DNA Library Prep Kit for Illumina (New England Biolabs) according to the manufacturer's instructions. A mock PCR was done to fine-tune the number of cycles needed for library amplification (7 cycles). Before sequencing, the size distribution of the libraries was assessed with an Agilent 2100 Bioanalyzer (Agilent Technologies). The in situ Hi-C libraries were sequenced with a 2 x 100 bp paired-end run on a HiSeq 2500 system (Illumina).

For preparation of UV-irradiated *P. vanderplanki* larvae, we first prepared anhydrobiotic specimens. Anhydrobiosis was induced as described previously with slight modifications: groups of eight last instar larvae were placed on pieces of filter paper imbibed with 0.44 mL distilled water in a glass Petri dish (65 mm diameter, 20 mm height) and five of these dishes were placed in a desiccator box (30 × 25 × 25 cm) containing 1 kg of silica gel (relative humidity < 5%). Larvae were then completely dried after 48 h in the desiccator. Wet and dry *P. vanderplanki* larvae were exposed to 254 nm UV radiation (100 mJ/cm^2) on a HL-2000 HybriLinker crosslinker (Analytik Jena, Jena, Germany). Groups of 40 wet larvae taken 1 h, 3 h and 24 h after UV exposure, groups of 40 dry larvae exposed to UV and taken 1 h, 3 h and 24 h after rehydration, as well as groups of 40 untreated wet larvae and 40 untreated dry larvae as controls, were homogenized in RNAiso Plus (Takara Bio, Kyoto, Japan), incubated for 5 min at RT, and then frozen at -30˚C. For RNA-Seq of frozen samples, total RNA was extracted with a Nucleospin RNA clean-up kit (Takara Bio) and a TruSeq RNA Sample Prep Kit -v8 (Illumina) following the manufacturers' protocols. The pooled library was sequenced using the Illumina HiSeq1500 system (1 biological replicate for each sample).

For the comprehensive transcriptome analysis, groups of Pv11 cells were subjected to various stresses. 5 × 10^6 Pv11 cells were resuspended in 2 mL IPL-41 medium and subjected to the following stresses: 0.5% (v/w) NaCl, 600 mM mannitol, 600 mM trehalose, 20 µM paraquat, and 42˚C heat shock. The time point where cells were first exposed to stress inducers were set as T0, and samples were then taken at 1 h, 3 h and 24 h. For heat shock, cells were exposed to 42˚C for 15 min before T0 and then sampled accordingly. Samples were prepared in triplicate. In brief, 4 × 10^7 cells were suspended in

2 mL preconditioning medium (10% FBS containing IPL41 medium: 600 mM trehalose at 1:9) for 48 h. These cells were transferred to a new microtube and collected at 400 g for 5 min. After removing the supernatant, cells were suspended in 400 µL of the pretreatment solution and 40 µL was placed in a 35 mm tissue culture dish. These cells were desiccated in a chamber (20 × 20 × 20 cm) set at ca. 5% relative humidity with 1 kg of silica gel and continuously desiccated for more than 10 days. For rehydration, 1 mL IPL41 medium containing 10% FBS was added to the desiccated cells and incubated for 1 h at 25˚C. Cells were gently resuspended in the same dish until sampling. At each time point, cells were collected by centrifugation at 800 g for 2 min (4˚C), and the culture medium was removed. Cells were suspended in 500 µL RNAiso Plus (Takara Bio), incubated for 5 min at RT, and then frozen at -30˚C. For RNA-Seq of frozen samples, total RNA was extracted with a Nucleospin RNA clean-up kit (MACHEREY-NAGEL, Düren, Germany) and a TruSeq RNA Sample Prep Kit -v8 (Illumina) following the manufacturers' protocols. The pooled library was sequenced using an Illumina HiSeq1500 system (3 biological replicates for each sample). For the Pv11 cell desiccation-rehydration time course (2), we used our previously reported data (3).

2. Cap trap RNA-Seq

Larvae were treated as described above. Pv11 cells were incubated in trehalose mixture (600 mM trehalose containing 10% (v/v) IPL-41 medium) at a density of 2×10^7 cells per mL for 48 h at 25°C, then collected as the T48 sample. For the T0 sample, 1×10^7 cells were collected before treatment. Groups of 10 wet larvae (wet, n=2), larvae desiccated for 24 h (D24, n=2) and over 48 h (D48, dry larvae, n=1) were homogenized in 600 µL of TRI Reagent LS (Molecular Research Center, Cincinnati, OH) and debris were discarded by centrifugation at 12,000 g for 5 min. Pv11 control samples (T0, n=1) and Pv11 desiccation samples (T48, n=1) were also resuspended in TRI Reagent LS. Total RNA was extracted from the supernatant using Direct-zol Miniprep Plus Kit (Zymo Research, Irvine, CA), following the manufacturer's instructions, including DNase I treatment. Extracted total RNA was submitted to CTR-Seq library construction following a previous study (4). Sequenced reads were demultiplexed with bcl2fastq, and were classified as either TSS, CDS, or TTS with in-house scripts depending on the adaptor sequences. rRNA reads were removed using tagdust2 (5) with human ribosomal DNA complete repeating unit (GenBank: U13369.1) as the query.

3. Genome assembly and gene prediction

To calculate heterozygosity within *P. vanderplanki*, we first submitted DNA-Seq data obtained in this study k-mer count with Jellyfish v2.2.4 (6) and profiling with GenomeScope v1.0.0 (7). Trimming of paired-end reads was performed with Trimmomatic tool v0.36 (8) using trailing quality clipping and adapter removal. Mate-pair reads were preprocessed using NxTrim tool v v0.4.1-0f17575 (9) with default parameters. K-mer analysis using the Kmergenie tool v 1.7016 with k=21 and diploid model approximation (10) was performed for genome size and coverage prediction.

We employed a meta-assembly to obtain a highly contiguous and complete genome assembly. All Illumina short reads were assembled with Platanus v1.2.4 (11) to remove heterozygous regions. This assembly was scaffolded and gaps filled in with PBJelly v 15.8.24 (-minMatch 8 -minPctIdentity 70 -bestn 1 -nCandidates 20 -maxScore -500 -noSplitSubreads --minMapq=45) (12). This assembly was further scaffolded with long-read assemblies in two iterations using the mate-pair reads with Metassembler v1.4 (bowtie2_maxins=11000 bowtie2_minins=4000 mateAn_A=5000 mateAn_B=10000) (13). The first assembly was obtained using a Hierarchical Genome Assembly Process (HGAP4) workflow (Pacific Biosciences, SMRT Link version 3.1.1) (14). HGAP4 consists of de novo assembly with Falcon, and assembly polishing with Arrow. Default parameters were applied, with the following exceptions: genome length was set to 150 Mb, the consensus diploid mode was set to true. The second assembly was obtained with the DBG2OLC pipeline (15) using our previous PacBio genome sequencing data (16) corrected with Spari (17) and Illumina short paired-end reads. In the first stage of the DBG2OLC pipeline, short but accurate contigs were created using SparseAssembler (two iterations, "LD 0 NodeCovTh 1 EdgeCovTh 0" for the first, "LD 1 NodeCovTh 2 EdgeCovTh 1" for the second; all Illumina PE and MP reads were used, including PE reads, resulting from NxTrim procedure). In the second stage, "AdaptiveTh 0.0001 KmerCovTh 2 MinOverlap 20" parameters were fed with the contigs file from the first stage and Spari-corrected PacBio reads in fasta format. The third stage (consensus calling) was run with the default parameters.

Additional scaffolding and gap filling were run for this meta-assembly with mate-pair reads using SSPACE v3.0 (18) and GapFiller v1.10 (19) tools. This assembly was further scaffolded with Hi-C reads with the 3D-DNA pipeline (20). Hi-C reads were processed with Juicer (21) software to obtain a contact matrix for visualization of the initial assembly with Juicebox (22) and to generate a list of deduplicated and filtered read pairs, which reflects the contact frequency between assembly fragments. This list (merged_nodups.txt) and the assembly.fasta file were applied to the 3D-DNA pipeline without a misjoin correction step. Regions of scaffolds containing visually detected misassemblies were corrected manually with Juicebox Assembly Tools (23). The resulting set of scaffolds was applied to the second round of read mapping and processing. In a subsequent step with 3D-DNA, automated scaffolding with two iterations of misjoin corrections was applied to the data. Final scaffold correction, unplaced scaffold ordering, and chromosome-size scaffold splitting were performed with Juicebox Assembly tools. The order of chromosomes was sorted based on their length, resulting in the Pv5.2 genome assembly.

The main statistical metrics of all assemblies were assessed using the Quast tool v5.0.2 (24). The completeness of assemblies was assessed using universal single-copy orthologues of Diptera species (3,285 proteins) with the BUSCO4 tool v4.0.6 or v4.0.2 (25). The DNA-Seq data from our previous study was downloaded from MidgeBase (http://bertone.nises-f.affrc.go.jp/midgebase), mapped to the genome with BWA mem v0.7.15-r1140 (26) and assayed with Qualimap v2.2 (27) after processing with SAMtools convert/sort v1.7 (28). GC ratio, gene density, DNA-Seq and RNA-Seq coverage were calculated with BEDtools v12.29.2-39-g5210e6f (29) for 10 or 50 kbp windows and visualized with Circos v0.69-8. Contaminant sequences were also assayed with BlobPlot v1.0 (30). The scaffold sequences of Pv5.2 and Pv0.9 were submitted to Diamond

BLASTX v0.9.24 (31) to search UniProt Reference Proteomes (downloaded 2017, Dec. 9th)(32) and then taxified with Blobtools. Taxification and coverage data were submitted to Blobplots to visualize the results.

In order to identify and classify repeat elements in genome scaffolds, we used RepeatModeler v1.0.10 (33) and RepeatMasker tools v4.0.7 (34). To this soft-masked genome sequence, we mapped all RNA-Seq data with the Hisat2 tool v2.1.0 (35). The merged bam file with all alignments was used as input to the BRAKER1 pipeline v1.9 (--softmasking 1)(36) using the soft-masked genome assembly. The resulting set of BRAKER1-predicted protein products were functionally annotated with InterProScan pipeline v5.26-65.0 (37). tRNA and rRNA genes were predicted with tRNAScan-SE (v1.3.1)(38) and barrnap (v0.6)(39). These gene predictions were merged into the initial gene set Pv5.2.0. Additionally, the nucleotide sequences of ARId sequences obtained from GenBank were submitted to minimap2 v2.17-r941 (40) or BLASTN v2.2.22 (41) to search against the genome or predicted coding sequences, respectively. MCScanX vMar312011 (42) was used to detect collinear blocks using blastp results (*E*-value < 1e-15) with default settings. Collinear blocks were visualized with synvisio (43).

We also scaffolded our previous genome assembly Pv0.9, which used DNA-Seq data from only insect specimens. The Pv0.9 genome assembly was aligned to the Pv5.2 assembly with D-genies (44) and produced a “pseudo-chromosomal” assembly (Pv0.9p). The Pv0.9 gene predictions were mapped to Pv0.9p with Liftoff (45). This Pv0.9p assembly was subjected to synteny analysis with Pv5.2. Amino acid sequences were subjected to round-robin Diamond Blastp search (--sensitive -k 0 -e 1e-15) and collinear blocks were detected with McScanX with a stringent threshold (-m 5) (31,42). The resulting collinear blocks were visualized with Synvisio.

<u>4. Analysis of CTR-Seq data</u>

TSS/mRNA/TTS tags from CTR-Seq were mapped to the genome with Hisat2 v2.1.0 (35) and were submitted to the HOMER2 v4.10.0 (46) analysis pipeline for TSS/TTS peak identification. Briefly, tag directories were constructed from mapped BAM files, and each file was submitted to findcsRNATSS.pl to identify tag peaks. Peaks within 50 bp of each other were merged into a single peak cluster with mergePeaks.pl. Identified peak clusters were annotated/quantified by annotatePeaks.pl. Raw peak cluster tag counts (CPM; counts per million) were normalized using UMI sequences by reducing sequence tags with the same UMI mapped to the same position. Sequence tags from coding regions (mRNA) were pseudo-mapped and quantified (TPM; transcripts per million) against the BRAKER coding sequences with Kallisto 0.44.0 (47). The findMotifsGenome.pl tool was used to identify motifs enriched close to TSS and TTS peak clusters.

Raw CTR-ONT reads were mapped to the genome with minimap2 v2.17-r941 (40) and were collapsed with TALON v5.0 (48). New gene predictions were compared with the BRAKER gene model with SQANTI3 v1.0.0 (49) and validated for completeness with BUSCO4 (v4.0.2, Insecta, Diptera). The mapping ratio of mRNA-Seq obtained during CTR-Seq was also calculated with Hisat2 v2.1.0 (35) and Qualimap2 v2.2 (27). Anti-sense, non-coding transcripts, and fusion genes were removed from the gene set. For

comprehensive identification of full-length non-coding polyA transcripts, we first removed ONT and Nextera Transposase adaptors by Porechop v0.2.3_seqan2.1.1 (50) and fixed strandness by adapter orientation (validated ONT reads). We then conducted a transcriptome assembly using paired-end mRNA-Seq data from our previous study (16) and the validated ONT cDNA-Seq reads with rnaSPADES v3.14.1 (51). This transcriptome assembly and the validated ONT reads were mapped to the genome with minimap2 v.2.17-r941(40). The output BAM files were merged and sorted with SAMtools v1.7 (28) and converted into BED12 format and clustered with bedtools2 v2.29.2-g5210e6f (bamToBed, merge) (29). Validated ONT reads and assembled transcripts in each cluster were obtained from the minimap2 output and converted into fastq files by SAMtools. These read clusters were subjected to 1-3 rounds of Racon v1.4.13 (52) polishing and consensus sequences were obtained by medaka v0.11.5 (53). Consensus sequences were mapped to the genome with GMAP v2017-11-15 (54) and validated by overlap with any BRAKER genes using BEDtools v2.29.2-g5210e6f (29). TSS and TTS tags were mapped onto these final transcripts with BWA mem v0.7.15-r1140 (26) and the orientations of mapped tags were used for transcript strand correction. Additionally, these transcripts were searched using BLASTN for homologs in the following dipterans (downloaded from EMSEMBL Metazoa on 2020/9/4) and *Polypedilum*; *Aedes aegypti* LVPAGWG (AaegL5), *Anopheles darlingi* (AdarC3), *Anopheles gambiae* (AgamP4), *Belgica antarctica* (ASM77530v1), *Culex quinquefasciatus* (CpipJ2), *Drosophila melanogaster* (BDGP6), *Glossina morsitans* (GmorY1), *Lucilia cuprina* (ASM118794v1), *Lutzomyia longipalpis* (LlonJ1), *Megaselia scalaris* (Msca1), *Musca domestica* (MdomA1), *Phlebotomus papatasi* (PpapI1), *Stomoxys calcitrans* (ScalU1), *Teleopsis dalmanni* (Tel_dalmanni_2A_v1), *P. pembai* (GCA_014622435.1), *P. nubifer* (Pn_0.9). These final non-coding genes were merged with the TALON-derived genes and manual curation resulted in the final gene set (Pv5.2.4). mRNA, CDS and amino acid sequences were obtained with the GFFread utility in Cufflinks v2.2.1 (55). Amino acid sequences were annotated by BLASTP v2.2.22 search against Swiss-Prot (downloaded on 2019/2/15)(41) and InterProScan v5.46-81.0 (37). TSS/TTS peak clusters, mapping results of sequencing data and the final gene set were visualized in the JBrowse environment for manual inspection. Candidate genes of horizontal transfers were detected by methods used to detect horizontally transferred genes in bdelloid rotifers (diamond blastp: --sensitive --index-chunks 1 -k 500 -e 1e-5; diamond_to_HGT_candidates.pl: --taxid_skip 54970 –taxid_ingroup 6656) (31,56).

Using the new gene set, we reclassified TSS/TTS peaks using in-house Perl scripts and validated ONT reads. In brief, ONT reads were mapped to the genome and transcript sequences with minimap2 v2.17-r941 (40). For each ONT read that mapped to a transcript, the genomic start and end positions were calculated using the pysam utility. The corresponding TSS/TTS peak cluster was calculated for each transcript using the start/end position of mapped ONT reads. If no ONT reads mapped to a transcript, the TSS/TTS peak with the highest HOMER score within 1,000 bp upstream/downstream of the mRNA start/end position were designated as the TSS/TTS peak cluster.

5. Variant calling

To estimate of level of intra-species genomic diversity , we estimated single nucleotide variants and indels using our previous genome resequencing data of six populations of *P. vanderplanki* within Nigeria (57). Pool-Seq reads were mapped to the *P. vanderplanki* genome with bwa-mem software v.0.7.10 (26). Resulting files were converted and cleaned of duplicated reads with SAMtools v1.9 and Picard 2.16.0-SNAPSHOT(58). Variant calling was performed with SAMtools mpileup and bcftools call v1.9 and filtered with GATK v4.1.20 with "DP < 10 || QUAL < 20" (28,59,60). The PoPoolation2 software tool (61) was used to measure allele frequency differences between populations and to estimate population differentiation (measured by the fixation index, *Fst*) along the genome with a sliding window approach (50 kbp windows). Selective sweeps were found by Pool-hmm (62) with k value = 0.001. To detect large-scale indels, we mapped PacBio reads of Pv11 cells and inbred insects to the genome with BWA mem (-x pacbio -M) and structural variants were Identified with Sniffles v1.0.12 (63).

We additionally reanalyzed Pool-Seq data and high-coverage DNA-Seq data of Pv11 cells and insect specimens (16) for generation of a phylogenetic tree to determine the genetic distances between populations. Each DNA-Seq data were mapped to the Pv5.2 genome using with BWA mem v0.7.10-r789 (26) and output converted into a sorted bam file with SAMtools v1.11-5-g0920974. Variant calling was performed with SAMtools mpileup and bcftools call v1.11-13-g78003de (Li, 2011; Li, et al., 2009) and filtered with GATK pipeline v4.1.19 with options "DP < 10 || QUAL < 20" (28,59,60). Consensus sequences were produced with bcftools consensus using both SNVs and Indels. From these sequences, a phylogenetic tree was built using alignment-free distance-based procedure by JolyTree software v2.1.211019ac (64).

All detected variants (SNVs, INDELs) were annotated for their effects on coding regions with snpEff v5.0c (65) using the Braker2 based gene predictions as the database.

6. Data acquisition for Diptera comparative genomics

For the chromosome level conservation analysis, we obtained dipteran genomes with >85% BUSCO completeness (Diptera lineage, **Additional Data S10, S11**). *Apis mellifera* and *Atta cephalotes* were used as outgroups. Coding and amino acid sequences for these organisms were downloaded from ENSEMBL Metazoa or NCBI Genbank (66). Additionally, coding sequences and amino acid sequences of *P. nubifer* and *Parochlus steinenii* were downloaded from Midgebase (16) and the GigaScience data server (http://gigadb.org/dataset/100256) (67), respectively. The genome and gene predictions of *P. pembai* were kindly provided by the authors of the previous study (57). The GC ratio of each genome was calculated with BBMap stats.sh (68).

We performed gene prediction for the genomes of *Chironomus tentans* (GCA_000786525.1) (69) and *Chironomus riparius* (GCA_001014505.1) (70). All genomes were submitted for BUSCO evaluation using the –long option to create an Augustus gene model. The gene prediction model in the output was used to predict genes for both species using the autoAugPred.pl script in the Braker1 suite.

The transcriptome assemblies of *Chironomus piger* (71), *Cricotopus albitarsis* (72), *Cricotopus draysoni* (73), *Cricotopus parbicinctus* (72), *Podonomus* sp. (74), *Procladius villosimanus* (74), *Trissopelopia nemorum* (74), *Paraheptagyia tonnoiri*, *Telmatogeton pectinata* (74), *Polypedilum nubifer* (74), *Cardiocladius* sp. (74) and *Kiefferophyes invenustulus* (74) were obtained from NCBI (**Additional Data S11**). Each transcriptome assembly was validated for completeness with BUSCO (Dipteran lineage). All assemblies had BUSCO completeness scores below 70% (19.2-58.2%); therefore, we performed reassemblies with Trinity v2.9.4 (default options) to see if more recent assemblers could improve the completeness. The associated SRA submissions for the transcriptome assemblies, *Chironomus columbiensis* (75) and *Chironomus dilutus* (76), were obtained from NCBI SRA with sra-tools v2.9.2. We observed improvements in *C. columbiensis*, *C. dilutus, C. draysoni*, *T. pectinata*, *Cardiocladius* sp. and *K. invenustulus* (72.6%-88.1% complete). Expression values were estimated with RSEM in the Trinity utility. The isoform with highest expression was used in the following analyses. ORF regions and amino acid sequences were predicted using TransDecoder v5.5.0 (Predict: --single_best_only).

To test whether the conservation ratio decrease in *P. vanderplanki* Chromosome 4 was not an artifact from different methods used to predict genes, we conducted *ab initio* gene predictions for all of the genomes we assayed. We submitted each genome to repeat masking with RepeatModeler v2.0.2a (-engine ncbi) and RepeatMasker v4.1.2-p1 (-xsmall -html -gff -xm). The resulting soft-masked genome was submitted to Braker2 v2.1.6 gene prediction using "proteins of any evolutionary distance". The protein file odb10_arthropoda was obtained and collated from OrthoDB (Downloaded at Dec 24, 2021). The genome, longest isoform sequence from published predicted proteome and the *ab initio* predicted proteome were submitted to BUSCO5 v.2.2 completeness validation (diptera_odb10, version 2020-08-05).

7. Comparative genomics of Diptera

The longest isoforms for each gene were used in the subsequent analyses. Diptera (from NCBI or ENSEMBL) and *Polypedilum* (*P. pembai*, *P. nubifer*, and *P. vanderplanki*) genomes were submitted to Orthofinder v2.4.1 for ortholog cluster construction. GC ratios for single copy genes were calculated with custom scripts. Gene ontology enrichment analysis with GOstat v2.52.0 was conducted for each clade-specific ortholog cluster (77). For 1-to-1 ortholog detection, amino acid sequences of *P. vanderplanki* and either *D. melanogaster* or *A. aegypti* were pooled and submitted to diamond blastp to determine bidirectional blast hits with custom scripts. Essential genes of *D. melanogaster* were obtained from Online GEne Essentiality (78) and the locations of ortholog genes were identified using the bidirectional blast hit ortholog information. The location of BUSCO genes was obtained from the default BUSCO4 output. We validated enrichment of essential gene orthologs or BUSCO genes with Fisher's exact test with the FDR correction for multiple tests (left side testing to detect depletion). Additionally, pooled amino acid sequences of all 51 Diptera species were submitted to diamond blastp search; gene pairs with bidirectional hits were designated as homologs. The numbers of conserved genes were counted for each chromosome of *P. vanderplanki*, *D. melanogaster*, *A. aegypti*, and *A. gambiae*, and the conservation ratio was calculated

by the number of genes shared / number of genes on that chromosome. Similar analyses were conducted with the Pv0.9p gene predictions and *ab initio* gene predictions; we used single directional best hits for Pv0.9p and bidirectional hits for *ab initio* predictions to determine homologs. The conservation ratio was calculated as previously stated.

For collinear block detection, amino acid sequences of *Chironominae* (in pairs; *P. vanderplanki* and *P. pembai*, *P. vanderplanki* and *C. tentans*, *P. vanderplanki* and *C. riparius*), Chironomidae (in pairs; *P. vanderplanki*, *B. antarctica*, *C. marinus*), and Culicomorpha (pooled; *A. aegypti*, *A. gambiae*, *P. vanderplanki*) were submitted to diamond blastp and the collinear blocks were detected with McScanX with default settings (42). Genomic loci with more than five genes with a maximum gap of 25 genes were determined as collinear blocks. Collinear blocks were visualized with synvisio (43).

To calculate the dN/dS values for *P. vanderplanki* and *P. pembai* orthologs, we determined 1-to-1 orthologs from bidirectional best hits using round-robin Diamond BLASTP search results (*E*-value < 1e-15). 1-to-1 orthologs were aligned with mafft v7.427 and converted with PAL2NAL v14 (79) to create corresponding codon alignments of each orthogroup. dN/dS ratios were calculated with codeml in the PAML package v4.9 (80).

8. Comparative transcriptome analysis

To identify transcripts that were differentially expressed in control and stress-induced Pv11 cells, we mapped the mRNA-Seq reads to the coding sequences with RSEM v1.2.30 (81) and Bowtie2 v2.3.5.1 via the Trinity utility v2.9.1 (82). Mapped counts were subjected to DESeq2 v1.26.0 (83) and edgeR v3.28.1 (84) analysis. Transcripts that were differentially expressed in control and stress-exposed (42˚C, ROS, trehalose, NaCl, mannitol) samples at the same time point were designated differentially expressed genes (DEGs, FDR < 0.05, FC>2)). For the trehalose preconditioning (PreCondTre)/rehydration time course, transcripts that were differentially expressed compared to the previous time point were designated DEGs (FDR < 0.05, FC>2). PCA analysis of all transcripts were conducted in R. Expression profiles of all DEGs were clustered by the ward method using the Spearman correlation in R. To determine pathways that were induced in each cluster, we performed gene ontology enrichment analysis using GOStat v2.52.0 (*p*-value<0.05, testDirection = “over”)(77). Gene ontologies with only one observation were removed. Enriched gene ontologies were categorized with Revigo (small option, accessed on 2020/9/10) (85). We then submitted TSS loci associated with each DEG to motif enrichment analysis using the HOMER2 suite. Each cluster was analyzed for motif enrichment in the -2000 to +150 bp region around TSS sites using findMotifsGenome.pl.

9. Data Availability

The genome, DNA and CTR-Seq sequencing data and annotations have been uploaded to NCBI under the accession ID PRJNA660906. The transcriptome sequencing data have been uploaded to GEO under the accession ID GSE158443. This Whole Genome Shotgun project of *P. vanderplanki* implemented with the Braker gene

prediction (v5.2) has been deposited at DDBJ/ENA/GenBank under the accession JADBJN000000000. The version described in this paper is version JADBJN010000000. We have established a dedicated webpage at MidgeBase2 (https://www.midgebase.org) hosting a Jbrowse genome browser (86). The v5.2.4 gene models and raw data used for figures can be accessed from this page (https://github.com/Kikawada-Lab-UT-NARO/Pvanderplanki_chromosomal_genome). Sequence manipulation and statistics, isoelectric point calculation was conducted with the G-language Genome Analysis Environment v.1.9.1(87,88) and R project.

ADDITIONAL TEXT

S1. A chromosome-level genome assembly of *P. vanderplanki*

To assemble the genome of *P. vanderplanki*, we used multiple DNA-Seq data from Illumina short reads and PacBio long reads, produced from the Pv11 cell line. Since the anhydrobiosis-capable Pv11 cell line is easy to culture in large quantities compared to insect specimens, we have been establishing genetic toolkits using this cell line (89-91). Anticipating the needs for Pv11 as a resource for anhydrobiosis research, we submitted the Pv11 cell line to genome and transcriptome sequencing using multiple sequencing instruments and technologies.

Three types of Pv11 DNA sequencing data (154M Illumina paired-end (PE) read, 85M Illumina mate-pair reads and 1.17M SMRT PacBio long reads) were used for the genome assembly. Mate-pair libraries consisted of three libraries with insert size 5-6 kb (21.6M pairs), 6-7 kb (24.8M pairs) and 8-10 kb (38.9M pairs). SMRT sequencing results included 1.17M raw reads (Average length 10,021bp, N50 length 14Mbp) and coverage corresponding to approximately 100x coverage of the predicted genome size. K-mer analysis of the Illumina PE reads resulted in a predicted genome size of 112 Mb, with 375x coverage. Profiling of the DNA-Seq data with GenomeScope (7) indicated the genome size to be 102.3-110.4Mbp, with 0.78-1.14% heterozygosity (SRR12736659: 0.78%, SRR12736662: 1.14%, SRR12736661: 0.78%, SRR12736660: 0.769%). Heterozygosity predicted from our previous high-coverage Illumina based DNA-Seq data (16) for insects was slightly higher (0.957%), suggesting a similar heterozygosity profile between insects and Pv11 cell line.

Although the Illumina PE-only assembly (Platanus + PBJelly) gave the best completeness (96.0%), the HGAP4 assembly derived from PacBio reads alone had a 12-times greater N50 length (2.2 Mbp) and the lowest scaffold count (401) (**Additional Data S1**). The hybrid DBG2OLC assembly yielded moderate statistics. The Platanus assembly scaffolded with mate-pair reads resulted in an increase in N50 (to 627 kb) with a slight decrease in scaffold number to 81,374. However, as a result of the scaffolding procedure, the N content increased (to 2910.2 per 100 kb). Thus, we sequentially scaffolded the scaffolded Platanus assembly with the HGAP4 and DBG2OLC assemblies (**Additional Data S1**), resulting in an assembly of 581 scaffolds, 390 of which were more than 10 kb long. The N50 length of this new meta-assembly was 658,565 bp with a BUSCO completeness score of 95.9%. This suggested that the meta-assembly was more

comprehensive than the three initial assemblies. We further scaffolded the meta-assembly with Hi-C reads (two libraries, each of 29 x 10^6 and 26 x 10^6 reads, were merged) using the 3D-DNA pipeline and subsequent curation with Juicebox Assembly Tools (**Additional Data S2, S3**), resulting in a final 118.9 Mb assembly (Pv5.2): 98.8% of the genome assembly is contained in the four largest scaffolds, possibly corresponding to four chromosomes, consistent with our previous karyotyping studies (**Figure 1a**). We were able to link two chromosomes determined by karyotyping (I~IV) with the superscaffolds (chr_1 ~ chr_4) in the genome assembly by scaffold size (IV/chr_4 is the smallest) or the paracentric inversion (III/chr_3) indicated below.

The final meta-assembly Pv5.2 comprised 388 scaffolds with an N50 length of 35.2 Mb, which equals the size of Chromosome 2 and a high BUSCO completeness score (95.7% complete, 1.0% duplicated, 3.6% missing, Diptera lineage). The previous Pv_0.9 assembly had BUSCO completeness score of 92.9% (C: 92.9%, [S:91.9%, D:1.0%], F:2.7%, M:4.4%, Diptera lineage), thus, the new Pv5.2 assembly had an increase of approximately 3% in completeness. This score is slightly lower than that of other mosquito's chromosome level genome assemblies. The increase in missing category may be due to two factors: (1) gene mispredictions during *ab initio* Augustus prediction (in both Braker and BUSCO4 runs), as our results are compared with highly curated genomes (2) possible intensive gene loss or mutation accumulation in *P. vanderplanki*, as we state in the following sections. The average number of ambiguous nucleotides was 518.4 per 100 kb, which was a marked improvement on the Pv0.9 assembly of 4,167 (16). There are 648 gaps with unknown length. Quality analysis using Blobtools indicated minimal contamination in Pv5.2, whereas Pv0.9 contained several bacterial contaminations (**Additional Figure S1ab**). A direct comparison showed that 77.35% (7,042 / 9,104) of the scaffolds of our previous genome assembly mapped to the new genome. Those that did not map were shorter than 30 kb (longest 28.5 kb, average length 1.2 kb, N50 length 1,398 bp). Only 140 of these non-mapped scaffolds were classified phylogenetically as Arthropoda or Nematoda from our Blobtools analysis, while 277 scaffolds matched with bacterial or fungal genomes and 1,654 were undetermined. This suggests that these scaffolds derived either from contaminants or genome assembly artifacts. We did not observe large differences in DNA-Seq or RNA-Seq coverage (**Additional Figure S1cd**). These statistics indicate that this genome assembly had greatly improved contiguity, completeness and quality compared to our previous genome assembly.

This chromosome scale assembly enabled the identification of putative centromeric regions. We identified 28 regions comprising a single repeat unit of approximately 145 bp and linker sequences of variable length (**Additional Data S4**), with 16 of these regions having >500x PacBio read coverage. All chromosomes had a high coverage of repetitive regions (1-11, 2-2, 3-4, 4-2). The longest region on Chromosome 1 (1-11: 36,597,727-36,620,193) had 78-fold higher coverage than the average for the whole genome, suggesting that it represents a 1.74 Mb region that collapsed into 22 kb during assembly. The centromeric repeat unit identified in *Chironomus pallidivittatus* centromeres has similar lengths (92), but the sequences itself had no similarity. Additionally, Hi-C contact maps did not indicate a RABL organization found in *Aedes* (20) and the positions of these loci did not match those found by karyotyping studies (93). Together, these loci would be the subject of further validation by methods such as

FISH. Similar, the telomere repeats in *Chironomus* were not found in this assembly (94), indicating a different telomeric repeat unit in *Polypedilum*. Unlike ceteromeric regions, we see indications of telomere-telomere interactions in Hi-C contact maps, supporting the existence of telomeres.

S2. Comparison with the *P. vanderplanki* genome (Pv_0.9)

It is well known that cell lines are prone to genomic changes, *i.e.,* whole genome duplication, accumulation of new chromosomes, and intensive mutations (95). The 50-year-old C6/36 cell line derived from *Aedes albopictus* has been found to contain large differences between other mosquito assemblies (96). Most of the DNA sequencing data used for genome assembly was obtained from the cell line Pv11, thus similar genome structural changes may have occurred. Since the initial establishment in 2002, the Pv11 cell line has been cultured for about 5 years before creating freeze stocks, corresponding to roughly 2,000 generations. Although such long cultures may cause structural variants, we assay cultures for changes in growth speed and desiccation tolerance capabilities on regular basis and restart cell cultures from freeze stocks when we detect changes, hence minimizing the possibility of large-scale genomic changes.

As a general comparison, the Pv5.2 genome assembly size is in line with our previous flow cytometry-based genome size predictions (97) and DNA and RNA sequencing data showed uniform coverage at between all chromosomes (**Figure 1a, Additional Figure S1cd**), thus denying large-scale genome duplication events. We also see that chromosome size ratio is in line with the karyotype data (Chr 1 = Chr 2 > Chr 3 >> Chr 4)(93), supporting that there are not large-scale chromosomal translocations.

To validate whether sequencing cell lines affects our analysis, we compared the genomic content of Pv5.2 with Pv0.9, which used DNA-Seq data only from insect specimens. We used our previous Illumina-based DNA-Seq data of inbred *P. vanderplanki* strain NIAS01 and the Pool-Seq data of wild *P. vanderplanki* populations to identify differences between Pv11 and each insect population. Initial analysis with the NIAS01 Illumina DNA-Seq data indicated that the mapping ratio to the Pv5.2 genome (97.63%) was higher than the mapping ratio to Pv0.9 (97.22%). The average mapping quality against Pv0.9 (53.8669) was higher than Pv5.2 (45.0091), indicating slight differences between Pv5.2 and Pv0.9. Indel detection with the PacBio reads from insect specimens also did not suggest large scale insertions or deletions (**Additional Data S9**)(16). We note that the quality of the Pv11 PacBio DNA-Seq data exceeds that of *P. vanderplanki*, therefore there is a possibility that long range indels may have not been detected in insect data. Phylogenetic analysis of consensus sequences of wild populations, the inbred NIAS01 insect line, and the Pv11 cell line constructed from SNVs suggested that the genetic distance between Pv11 cell line and other wild or NIAS01 strain was within inter-population diversity (**Additional Figure S2a**), therefore the Pv11 genome may be interpreted as one population of *P. vanderplanki*. We hypothesize that both Pv11 and NIAS01 has different selection pressures compared to that of wild populations, which may explain the unique differences from wild populations.

Additionally, to determine whether the Pv11 and insect genome differs at gene level, we scaffolded the Pv0.9 assembly to chromosomal level based on the Pv5.2 assembly to

produce a “pseudochromome” assembly (designated Pv0.9p). Initial whole genome alignment between Pv0.9 and Pv5.2 indicated minimal inversions and translocations (**Additional Figure S2b**). The latter half of Chromosome 3 was highly fragmented in the Pv0.9 assembly, suggesting this region may be enriched in sequences that make genomic assembly difficult (**Additional Figure S2b**). Previous attempts in chironomids have resulted in fragmented genome assemblies (16,57,67,69,70), possibly these repetitive sequences may have interfered (98,99). We observed slightly lower GC ratio in Pv0.9p (Pv0.9p: 28.30%, Pv5.2: 28.09%), possibly due to repeat elements being correctly assembled in the Pv5.2 assembly. Similar chromosome-wide decreases in GC ratio were observed in Chromosome 4 (**Additional Figure S2c**). Comparison of gene loci between Pv0.9p and Pv5.2 did show several inversions and translocations, however majority of the genes showed high conservation of synteny (**Additional Figure S2d**).

Together, we propose that the genome of Pv11 obtained this study does not differ greatly with the genome of *P. vanderplanki*, thus this genome would be compatible with comparative analysis with other dipterans.

S3. Gene prediction

Several samples of RNA sequencing data were used for the gene prediction pipeline. They included previously sequenced reads of RNA derived from different developmental stages (male and female adults, larvae, and pupa, 44M reads) of *P. vanderplanki* as well as larval RNA samples from different stages of the desiccation-rehydration cycle (186M reads) (3,16). We also used newly sequenced short reads derived from larvae and a *P. vanderplanki* cell line (Pv11 cells) after treatment to various stress conditions (547M reads, NaCl, mannitol, trehalose, paraquat, and heat shock, UVC exposure). Gene prediction with BRAKER1 yielded 17,852 potential protein-coding genes and 19,117 transcripts, which had a BUSCO completeness score of 95.8%. Of these transcripts, 18,179 and 10,674 mapped to the previously predicted gene set and the Swiss-Prot database, respectively. Chromosome 4 contained nearly twice as much protein coding genes / Mbp but only half in non-coding genes (**Table 3, Additional Figure S1ef**)

We identified 31 *Lea* orthologs, an increase of four copies compared to our previous studies (16). *PvLea12* and *PvLea9* were tandemly duplicated, forming a tandem unit arranged as (*PvLea12a*/g17258 and *PvLea9a*/g17259) and (*PvLea12b*/g17260 and *PvLea9b*/g17261). It is possible that the increase in *Lea* orthologs results from a gene duplication that occurred after the establishment of Pv11.

S4. Application of CTR-Seq to *P. vanderplanki*

Using the TSS/TTS tags from the CTR-Seq data, we predicted 21,805 and 24,536 TSS and TTS peaks, accordingly (**Additional Data S5, S6, Additional Figure S3a**). This corresponds to average of 1-2 peaks for each gene. Automatic annotation of TSS peaks classified 58% of called peaks as locating in the 5'-UTR regions (12,682) associated with 8,838 genes (8,893 transcripts), A further 18% were located in exonic (1,486) and intronic (2,429) regions. The remaining 24% were located in 3'-UTR (1,405) and intergenic regions (3,791). In contrast, 40% of TTS peaks were located in 3'-UTR

regions, corresponding to 6,599 genes (6,617 transcripts). Similar to TSSs, 20% were located in exonic (2,464) and intronic (2,414) regions, while 40% were not associated with TSSs (5'-UTR: 6,215; intergenic: 3,565). Visual inspection of mis-annotated peaks in Jbrowse suggested the existence of transcripts that were not predicted in the BRAKER1 gene set. UMI-corrected tag counts of TSS and TTS peaks were compared with the gene expression values of the associated genes quantified by mRNA-Seq (**Additional Figure S3b**). We did not test for significant differences since these libraries were sequenced in single or double replicates.

To test whether the called peaks were truly TSSs and TTSs, we conducted motif enrichment analysis in the -2000 bp to +150 bp regions around TSS peaks and in the -500 bp to +500 bp regions around TTS peaks. A total of 437 known motifs were enriched in these regions (**Additional Figure S3cd**). Core promoter motifs were included in this list, *i.e.*, the Drosophila Initiator motif (-TCAGT-, q-value < 0.0001, 92.88% of TSS) and the TATA box motif (-TATAA-, q-value<0.0001, 16.09% of TSS). The TATA-box motif was found in fewer transcripts than the Initiator motif, which may be an artifact of the low GC content of the *P. vanderplanki* genome. This may also imply that transcription initiation sites that do not rely on a TATA-box may be in the majority; high levels of AT within the genome would cause misrecognition of core promoter sites. On the other hand, we observed enrichment of the upstream U-rich motif, the polyA motif, and the downstream A-rich motif around TTS peaks; over 60% of target sequences contained the polyA and A-rich motifs.

The ONT cDNA-Seq data from CTR-Seq enables full-length sequencing of mRNA molecules. We collapsed these reads into splice variant structures using TALON (**Table 2**). Preprocessing raw ONT cDNA-Seq reads with either Canu v2.0 (100) or TranscriptClean v2.0.2 (101) or adaptor removal with Porechop (50) did not increase BUSCO completeness. The transcriptome predicted using non-processed raw reads with TALON had high BUSCO4 completeness, few Missing categories (Insecta and Diptera lineages) and a minimal decrease in mRNA-Seq read mapping ratio. This initial gene set comprised 44,152 genes (589,775 transcripts), of which 24,417 were novel genes. Comparison with the BRAKER1 gene model by SQANTI3 indicated that approximately 77% of the transcripts were antisense transcripts; removal of these transcripts had a minimal effect on BUSCO scores. Further curation (removal of erroneous genes, rescue of missing Braker genes) resulted in the Pv5.2.4 gene set composed of 18,989 genes (65,981 transcripts) with a BUSCO4 completeness score of 96.2% (Diptera lineage). Approximately 90% of the refined set of transcripts were annotated by InterProScan search.

We have previously experimentally validated the full-length sequence of several anhydrobiosis-related genes, mainly glutathione peroxidase and trehalose-6-phosphate synthase (TPS). Although we did not obtain the full-length sequence of glutathione peroxidase (AB872496.1) due to misprediction at the BRAKER1 gene prediction stage (the gene was split into two genes), we were able to obtain full length sequences of both TPS splice variants (AB490331.1). Two major heat shock factor (HSF) variants derived from graph-based gene prediction were found (102), including a transcript lacking the 5th exon.

We detected a total of 70 genes as possible horizontally transferred genes (0.47% of 14,773 processed or 0.39% of 17,898 all genes), consistent with our previous estimations of horizontally transferred genes within Diptera (103). Fifty of these genes were not-annotated (blastp against SwissProt, 1e-15 threshold). The remaining 20 genes included Zinc finger proteins, probable histones, lipid desaturase, etc.; no previously known anhydrobiosis genes were included in this list. There was no significant enrichment in Chromosome 4 (chr_1: 20; chr_2: 12; chr_4: 15, chr_4: 17 genes; p-value > 0.05, Fisher's exact test).

Noting that transcripts that were not predicted by BRAKER1 existed in the initial TALON gene set and that TSS/TTS locations supported the existence of these transcripts; we filtered the full-length ONT reads for potential candidates. Racon polishing and medaka consensus generation resulted in a total of 1,092 long non-coding RNA (lncRNA) genes. 291, 49 and one of these transcripts were conserved in *P. pembai*, *P. nubifer* and the Antarctic midge *B. antarctica* (BLASTN against genome, E-value threshold < 1e-30), respectively, while none were found in other dipterans. We observed several lncRNA genes upstream of protein coding genes, in particular within one of the strong constitutive promoters, the 121-promoter (91), implying that this lncRNA may contribute to the regulation of the downstream gene g7775 (**Additional Figure S3ef**).

Our results show that CTR-Seq can be applied to non-model organisms to overcome one of the key difficulties with genome annotation: how to effectively and correctly identify transcription start sites (TSSs) and terminal sites (TTSs) to allow analysis of regulatory elements, simultaneously with sequencing full-length transcripts.

S5. Variant analysis of the chromosome level genome.

We estimated genetic diversity using our previously sequenced genome data on six *P. vanderplanki* populations to identify regions that are differentiated between populations (**Additional Data S9**). SNVs detected had coverage in line with the average coverage of the whole sequencing data (**Additional Figure S4a)**. We identified 817,057 ~ 1,667,109 SNVs from each population, corresponding to 0.70-1.42% of the whole genome. Additional analysis using Illumina-based DNA-Seq data from the inbred line NIAS01 indicated similar numbers (1,477,274 variants, 1.26%). Using Pv11 DNA-Seq data showed lower numbers (0.56~0.61%), which we anticipated since we used these cells for genome sequencing. Approximately 7.47-10.89% and 17.32-20.42% of the SNVs were located on exonic and intronic regions, respectively. Pv11-DNA-Seq data indicated slightly lower values (approx.6.5-6.8% exonic, 11.2-11.3% intronic). The missense/silent mutation ratio was approximately 0.47-0.60% for insects (0.66-0.68 for Pv11). The slight increase of exonic SNVs may affect gene structure, as seen in the increase of missense/silent mutation ratio. Within the four chromosomes, Chromosome 4 had the lowest Ts/Tv values in all insect populations and Pv11 data. Additionally, we detected indels from PacBio data of insect specimens and Pv11 cells (**Additional data S9**), which suggested that indel profiles did not differ greatly between NIAS01 and Pv11.

We then analyzed the diversity between populations. Pairwise nucleotide diversity (π) between the Tashan nabai population of *P. vanderplanki* and all others shows increased π in the right arm (16.5~Mb) of Chromosome 3 and the region from ~3 Mb to

~12 Mb of Chromosome 4 (**Figure 1e**). Negatively correlated with π, the corresponding regions in Chromosome 3 and 4 have decreased *Fst* values in all pairwise comparisons (**Additional Figure S4b**). Such reductions in *Fst* can be explained by the high π observed, as indicated above. In comparison, the regions with low π or high *Fst* values in Chromosome 3 and 4 have increased numbers of selective sweeps (**Additional Figure 4c**). The increase pairwise nucleotide diversity in chr_3 may correspond to the paracentric inversion located in Chromosome 3 observed in previous karyotyping studies (93). A paracentric inversion can lead to alterations in gene expression, as well as chromosome anomalies during meiosis and reduced recombination (104,105). Reduced recombination arising in individuals with heterozygotic paracentric inversion is an interesting phenomenon that can result in an accumulation of beneficial or deleterious alleles in the local population (106). Such inversion strongly reduces gene flow and thereby protects regions of the genome from the homogenizing effects of gene flow (107,108). Regions containing paracentric inversions show relatively high nucleotide divergence between populations that are at an early stage of speciation due to disruptive selection (104,109). *Fst* values are low in the paracentric region, because intra- and inter-population nucleotide diversity in the inversion region is high (**Figure 1e, Additional Figure S4b**). Inversions can reduce recombination rates and are therefore regarded as a genetic mechanism for protecting sets of locally adapted genes and promoting ecological divergence between populations, which ultimately can lead to speciation. However, it is impossible to say whether this paracentric inversion has played any role in the adaptation of *P. vanderplanki* to anhydrobiosis or of chironomids to other environmental conditions.

S6. Ab initio gene predictions and transcriptome assembly for chironomids

The genome sequences of *C. tentans* and *C. riparius* are available at NCBI (69,70), but corresponding gene predictions were not. Therefore, we subjected both genomes to *ab initio* gene prediction using the Augustus model obtained during BUSCO validation. We predicted 17,975 and 18,797 genes for *C. tentans* and *C. riparius*, respectively. Both gene predictions had high BUSCO completeness scores (protein, Diptera lineage, *C. tentans* C:88%, F:3.7%, M:8.3%, *C. riparius* C:84.2%, F:5.0%, M:10.8%), close to that at the genome level (*C. tentans* C:90.6, F:3.2%, M:6.2%, *C. riparius* C:86.0%, F:4.4%, M:9.6%). A recent paper has reported a chromosome level genome assembly for *Propsilocerus akamushi* and suggested a four-chromosome genome (110). However, multiple karyotyping studies has suggested a three-chromosome genome (111-113), thus, we did not include this genome into our chromosome level synteny analysis.

Several studies have conducted transcriptome sequencing and assemblies on chironomids, but few of these assemblies have been released on publicly available databases (67,71-76,114,115). All assemblies had low completeness (C:19.2-58.2%) therefore we investigated whether more recent assembly methods provide better assemblies. We observed improvements (BUSCO completeness Complete > 80%) in *Chironomus columbiensis*, *Chironomus dilutus*, *Cricotopus draysoni*, *Podonomus* sp., *Trissopelopia nemorum*, *Paraheptagyia tonnoiri*, *Telmatogeton pectinata*, *Cardiocladius* sp. and *Kiefferophyes invenustulus*. We submitted these transcriptome assemblies for amino acid sequence prediction with TransDecoder using the most highly expressed

transcript for each gene. BUSCO scores after this process decreased to 59.2%-81.5%, but this still represented a marked increase over previous assemblies.

S7. Comparative genomics with Diptera

Since our initial analysis based on ENSEMBL and GenBank genomes were focused on a wider lineage range, we wondered whether how the conservation ratio would proceed within chironomids. We submitted 18 chironomid genome and transcriptomes (additionally two outgroup mosquito genomes) to the same analysis (**Additional Figure 5a**). We observed that the conservation ratio for Chromosome 4 was indeed lower for within chironomids. Additionally, the phylogenomic tree of 666 ortholog groups was consistent with phylogenetic relationships inferred in previous studies. Interestingly, the node height of *Chironomus* (0.0716) was lower than that of *Polypedilum* (0.1261), suggesting *Polypedilum* may have faster evolutionary speed than *Chironomus*. Using only genome assemblies gave similar results (*Polypedilum* 0.092, *Chironomus* 0.051, data not shown).

To ensure that our analysis was not a technical artifact from using Braker for predicting genes in *P. vanderplanki*, we conducted *ab initio* gene prediction using Braker2 (designated "*ab initio* gene set" below) for all 53 Diptera genomes obtained from ENSEMBL, NCBI, and chironomid genomes including *P. vanderplanki* (**Additional Data S12**). The number of genes predicted for each organism were relatively similar to the original gene models with several exceptions, e.g., >40,000 genes were predicted for *A. aegypti*, 3x compared to ENSEMBL gene set (**Additional Data S12**). We observed higher number of genes in chironomids compared to other Diptera, which may suggest a lineage specific increase in gene content. We observed both decreases and increases in BUSCO completeness, both possible due to (1) ENSEMBL registered gene models have been extensively validated (e.g., *D. melanogaster*, *A. aedes*, etc.) thus have ~100% BUSCO completeness (2) more recent gene prediction algorithms and databases may have improved the gene predictions. In conclusion, majority of the genomes show gene numbers typical of Diptera and high BUSCO scores, therefore we submitted these gene sets to conservation analysis as previously conducted.

First of all, we observed that the deduction of conservation ratio in *P. vanderplanki* Chromosome 4 (Pv-chr4) can be observed in this *ab initio* gene set as well. Secondly, we observe low gene conservation throughout the genome for *A. aegypti*, however the conservations ratio is consistent between the three chromosomes, therefore is a different feature from what we observed in *P. vanderplanki*. We presume that this is an artifact from false positive gene predictions as we predicted >40,000 gene for this species. Finally, we also observe large deductions in conservation in the Y and 4th chromosome of *D. melanogaster*. Interestingly, the 4th chromosome shows lower conservation in only Culicoidea (*e.g.*, *Anopheles*, *Aedes* species), suggesting a lineage specific loss of *D. melanogaster* Chromosome 4 (Dm-chr4) genes within Culicoidea. However, this would be out of the scope of our study, thus we do not discuss it in detail. We also observe reductions in *D. melanogaster* Chromosome Y in both the ENSEMBL and *ab initio* gene sets. While the reduction pattern is quite similar to Pv-chr4, Pv-chr4 harbors much more genes compared to Dm-chr Y (Pv-chr 4 > 2,000 genes, Dm-chr Y ~20 genes), thus a

direct comparison would not be inadequate. Together, the *ab initio* gene set shows similar results to what we see using published genomes, thus we conclude that our results are not biased from using different gene prediction methods.

We then submitted the Pv5.2 genome to synteny analysis with other chromosome-level mosquito genomes (Figure 2c) to understand the evolutionary origin of Chromosome 4 genes. The default settings of McScanX uses a low threshold. Chromosomes 1, 2 and 3 were observed to have counterparts in *A. aegypti* and *A. gambiae* (Pv1 : Aa-3p/1p, Ag2-R/X, Pv2: Aa-2q/2p, Ag-3R/2L, Pv3: Aa-1q3p, Ag-2R), including the arm breakage and inversion observed between *A. aegypti* and *A. gambiae* (20). On the contrary, Chromosome 4 lacked any synteny blocks with any other dipteran chromosomes, initially suggesting this chromosome may have been obtained after the divergence of chironomids and other mosquitoes.

We extended this analysis to the genomes of chironomids with four chromosomes (*C. tentans*, *C. riparius*), but we detected very limited, if none, synteny blocks with *P. vanderplanki* Chromosome 4 in either genome (**Additional Figure S5ef**). Both homolog and 1-to-1 ortholog detection indicated that 30-60% of Chromosome 4 genes are conserved in each species (*C. tentans*: Pv-chr 1: 3,546/5,275, Pv-chr 2: 3,062/4,911, Pv-chr 3: 2,597/4,197, Pv-chr 4: 1,182/3,241; *C. riparius*: Pv-chr 1: 3,509/5275, Pv-chr 2: 3,044/4,911, Pv-chr 3: 2,599/4,197, Pv-chr 4: 1,115/3,241, **Additional Figure S5a**). considering the conservation ratio calculated above and the loss of synteny, we hypothesize that the loss of synteny within Chironomids may be the result of extensive intra-chromosomal recombination or the fragmented genome assembly hindering correct synteny analysis. Further analysis of 1-to-1 orthologs (bidirectional best hits) indicated that genes on each chromosome are dispersed throughout other dipteran genomes (**Additional Figure S5g**, Ag=*A. gambiae*, Ag-chr X:38, Ag-chr 2L:145, Ag-chr 2R:168, Ag-chr 3R:114, Ag-chr 3L:156, total 633/3,241; Aa=*A. aegypti*, Aa-chr 1:136, Aa-chr 2:256, Aa-chr 3:261, total 661/3,241), supporting intra-chromosomal recombination creating this somewhat mosaic conservation pattern.

S8. Ortholog clustering

We performed ortholog clustering of *Polypedilum* protein sequences at three levels: high quality genomes obtained from ENSEMBL (Clustering 1), publicly available draft chironomid genomes (*C. tentans*, *C. riparius* and *Parochlus steinenii*, Clustering 2), and publicly available chironomid genome and transcriptome assemblies (*Podonomus* sp., *P. steinenii*, *T. nemorum*, *Paraheptagyia tonnoiri*, *T. pectinata*, *C. draysoni, C. marinus*, *Cardiocladius* sp., *B. antarctica*, *K. invenustulus*, *C. riparius*, *C. columbiensis*, *C. tentans*, *C. dilutus* and *Culicoides sonorensis*, Clustering 3).

We submitted 746,218, 159,429, and 818,365 protein sequences for OrthoFinder clustering, resulting in 33,807, 16,976, and 48,796 orthogroups for Clustering 1, 2, and 3, respectively. Approximately 90% and 60% of the genes were classified for genome-based and transcriptome-based clustering, respectively. Within these clusters, 1,014, 1,060, and 666 ortholog groups were used for species tree construction for each clustering, respectively. Each species tree reflected the phylogenetic relationships suggested in previous studies.

We screened the Clustering 1 result for *Polypedilum*-specific ortholog clusters. Seven out of the ten *Polypedilum*-specific orthogroups with the largest gene numbers (*e.g.*, conserved within at least one *Polypedilum* species, **Additional Data S16**) had InterProScan annotations related to transmembrane proteins, for which the ionotropic receptor domain was predicted in several cases. This list included the Lil protein family, a protein family conserved in only *P. vanderplanki* and *P. pembai*.

S9. Comparative transcriptomics of Pv11

Rational of conditions for transcriptome sequencing

The requirement for trehalose preconditioning in Pv11 cells suggests the presence of trehalose and/or osmotic stress may be an early initiator of successful anhydrobiosis (2). Previous studies have shown that larvae exposed to mannitol and NaCl accumulate trehalose at low and high levels, respectively (116). Therefore, exposing Pv11 cells to the same level of osmotic pressure from different sources should clarify whether treatment with trehalose is responsible for the changes observed during trehalose preconditioning. Additionally, water loss would cause cell shrinkage, *i.e.,* mechanical stress, and therefore stress sources that do not cause osmotic stress, such as heat or exposure to reactive oxygen species as a result of paraquat treatment, would make ideal negative controls for trehalose treatment.

Therefore, we submitted Pv11 cells to transcriptome sequencing after exposure to various stress mimicking the anhydrobiosis trajectory: heat shock (42˚C), paraquat (oxidative stress, (117)), osmotic stress with different osmolytes (NaCl, mannitol, trehalose), trehalose preconditioning to induce anhydrobiosis (PreCondTre) and the rehydration cycle.

We note that transcriptome analysis of Pv11 may not show the completely same cellular dynamics that occur in insect specimens, as insect bodies have highly differentiated tissues that show specific gene expression profiles. However, it is general that the cellular dynamics identified from cell lines show similar profiles with the organism of origin. Additionally, as we discussed in **Additional Text S1**, the Pv11 cell line may become a foundation for understanding cellular preservation. Understanding the response to various stresses in Pv11 would provide following studies a basis for expanding our understanding of cellular protection.

General analysis

To present an overview of the transcriptome profiles from multiple conditions, we submitted the expression matrix to principal component analysis. PCA analysis using the first three PC axis indicated two different trajectories between mechanical stress-exposed (*e.g.*, heat and paraquat) and trehalose-accumulating samples (*e.g.*, NaCl, Mannitol, etc., **Additional Figure 6a**). Conditions mimicking similar stages of the anhydrobiosis entry and recovery showed similar expression profiles: NaCl, trehalose, PreCondTre prior to 36 h; PreCondTre 48 h, 0 and 3 hpr; 12~ hpr. The initial expression profile of samples exposed to mannitol was similar to those exposed to NaCl and trehalose, but by 24 h mannitol samples showed a profile intermediate between those of 3 h and 24 h trehalose

or NaCl samples. These initial responses to NaCl, mannitol and trehalose (including PreCondTre) were very similar, suggesting that the first stage of trehalose preconditioning may be caused not by trehalose itself but by osmotic stress regardless of the stress source. In addition, late stages of preconditioning were also clustered, suggesting the transcriptional response in this point differs from that of the initial osmotic stress. The late stages of preconditioning also resembled the initial stages of recovery, implying that proteins required for the initial recovery (*i.e.,* trehalose catabolism) may be regulated during the late stages of trehalose pretreatment, *i.e.,* no effect of desiccation treatment. Samples exposed to heat or paraquat show similar expression profiles, but these are completely different from the profiles of samples that mimic anhydrobiosis.

PCA revealed that there are transcripts that contribute greatly to the difference between mechanical and trehalose-exposed conditions. Therefore, we examined the contributing rate of each transcript to the first three principal components. Transcripts positively contributing to both PC1 and PC2, negatively to PC3 comprised various anhydrobiosis-related genes, *e.g.*, those located in ARIds, namely LIL and LEA protein gene orthologs, and antioxidative stress proteins (catalase, thioredoxin, glutathione *S*-transferase; **Additional Data S17**). These suggest that LEAs, LILs, and anti-oxidative stress proteins comprise the majority of the stress response in Pv11. In contrast, transcripts contributing negatively to PC1 or PC2, positively to PC3 included those encoding various ribosomal proteins or heat shock proteins (sHSP, HSP70, HSP90), respectively. This was predictable, as chaperons would be required for heat shock response induced by the heat exposure. Furthermore, our previous transcriptome analysis has suggested various pathways related to protein expression are suppressed during entry into anhydrobiosis (118), thus the negative contribution of ribosomal proteins to PC1 and PC2 would be consistent with our previous data.

Clustering expression profiles and enrichment analysis

We then submitted the expression matrix to clustering and gene ontology enrichment analysis to identify biological processes regulated in each condition/time point. There were 22,747 transcripts with average TPM >1, of which 8,969 transcripts were differentially expressed. Differentially expressed transcripts formed eight clusters based on the Spearman correlation between expression profiles (**Additional Figure S6b**). Nearly one third ("Suppressed", Cluster 1 and Cluster 2) was highly expressed in control/heat/paraquat samples, indicating that these transcripts were immediately suppressed (within one hour) in trehalose-exposed/accumulated samples. These clusters were enriched in a variety of cellular processes (**Additional Data S18**). This finding is consistent with our previous transcriptome analysis of Pv11 cells (118). Other clusters were broadly categorized into three groups, *i.e.*, the initial stages of trehalose treatment ("Early", Cluster7), stages with long exposure to high concentrated trehalose ("Mid", Cluster 4 and Cluster 5) and late preconditioning stages to rehydration ("Late", Cluster 3 and Cluster 6). Cluster 8 genes were expressed in heat and ROS conditions. Each group were enriched in metabolic and antioxidative stress related gene ontology terms (**Additional Data S18**). Many of these biological processes have been implicated to contribute to anhydrobiosis.

Regulation of long non-coding transcripts

During our general analysis, we noticed several lncRNA genes identified by our CTR-Seq data to be significantly regulated. Several of these were located upstream of differentially expressed transcripts, implying that these non-coding genes may act as regulators for downstream protein-coding genes. In particular, the non-coding gene (nc472) located in the 121-promoter (91) was differentially expressed, together with its downstream gene g7775 (**Additional Figure S6c**). We have yet to determine whether these non-coding transcripts play any role in anhydrobiosis, but a high degree of correlation between the expression profiles of several non-coding and coding transcripts suggests the possibility that these lncRNAs may be enhancer RNAs.

Motif enrichment of promoter regions

The rich data obtained from sequencing approximately 30 conditions (each N=3) and the identification of transcription start sites from CTR-Seq has provided the starting point for identifying condition specific promoter elements through motif enrichment analysis of upstream sequences.

We observed enrichment of multiple motifs in the upstream sequences of genes encoding transcripts in each cluster. The 2,000 bp upstream of each TSS was enriched with motif related to HSF (g3611) and nuclear transcription factor-Y (NF-YA: g7210, NF-YB: g5893, NF-YC: g11889), previously identified as master regulators of anhydrobiosis in *P. vanderplanki* (3,89,102). The NF-Y binding motif was enriched in "Suppressed" gene clusters (mechanical stress-responsive Cluster 5), while heat shock elements (HSEs) were enriched in late trehalose treatment and early rehydration stages (Cluster 4 and 8). The HSF binding site HSE is enriched in upstream regions of anhydrobiosis-related genes, *i.e.,* LEA protein, protein L-isoaspartyl methyltransferase and glutathione *S*-transferase genes, suggesting that such genes are directly regulated by HSF. On the other hand, NF-Y is present in the upstream regions of genes encoding transcripts of various pathways. The expression of each transcription factor correlated well with the enrichment profiles (**Additional Figure S6d**). We have previously hypothesized that the suppression of NF-Y may reduce the energy demand on the cell as transcripts and proteins not required for anhydrobiosis will not be produced (3,119), which our current data is consistent with.

In summary, we hypothesize that osmotic stress and another unknown factor resulting from long exposure to high concentrations of trehalose may be two major, but distinct sources of transcriptional regulation, with the former acting before, and the latter after, a transition point somewhere between 12-24 h after trehalose treatment or accumulation. We have previously estimated the NFY-C transcription factor and Heat Shock Factor 1 (HSF1) to contribute greatly to Pv11 anhydrobiosis (3,89), and furthermore observed calcium signaling to be the starting point of a signal cascade (90), eventually leading to the regulation of genes required for anhydrobiosis. These data suggests that the calcium signaling triggered by osmotic stress may directly or indirectly

induces NFY-C signaling, which in turn regulates HSF1 for the regulation of various anhydrobiosis genes, however the link between each component remains to be identified.

S10. Subfunctionalization in the ARId1 locus

During the data analysis conducted in **Additional Text S9**, we observed several expression profiles trends in the ARId1 locus (**Additional Figure S6e**), containing numerous LEA and LIL protein coding genes. Our previous phylogenetic analysis has indicated closely located paralogs are genetically closer to each other compared to further loci (120). This suggests that these *Lea* paralogs may have been obtained through tandem duplication in multiple instances. Paralogous genes are commonly hypothesized to have functions slightly diverse from the ancestorial gene, therefore analyzing closely located paralogus loci may imply functional diversity/subfunctionalization between paralogs.

To validate this, we first used differential expression data to group consecutive genes into "Block" that had similar differential profiles. From the insights from the comparative transcriptome analysis, we used initial responses against osmotic stress, more specifically non-trehalose conditions (mannitol and NaCl) and trehalose exposed conditions (Trehalose and PreCondTre), to group the ARId 1 genes. Consecutive genes were grouped whether most isoforms from a single gene were differentially expressed in either non-trehalose conditions (Mannitol and NaCl conditions, (5) Mannitol T3; (6) Mannitol T24; (7) NaCl T1; (8) NaCl T3; (9) NaCl T24 in Figure 3d) or early Trehalose conditions (Trehalose T0vsT24/PreCondTre T0vsT12 in Figure 3d), or both. LEA protein coding genes are present in three Blocks (Block 1, 3 and 4) and Block 2 consists only of LIL protein coding genes (Figure 3a). Block 1 consists of 31 genes (g16168/*PvLil6* to g16197) to be regulated widely across the NaCl, mannitol, trehalose and PreCondTre datasets (Figure 3a). Block 2 was consisted of three genes (g16198/*PvLil11*, g16199, and g16200/*PvLil2*), regulated mainly in the PreCondTre and early rehydration samples. Block 3, comprising four genes (g16201/*PvLea17* to g16204/*PvLea4*), was regulated by NaCl treatment. The last Block 4 contained four genes (g16205/*PvLea5* to g16208/*PvLea11*) were regulated in the 24-h trehalose sample or between 0-12 h in the PreCondTre sample. This observation alone suggested that *Lea* and *Lil* genes within ARId1 has condition specific expression profiles. Interestingly, LEA orthologous proteins in most Block 3 and 4 (PvLEA7, PvLEA4, PvLEA5, PvLEA6, PvLEA8 and PvLEA11) form a single clade and contains increased numbers of the "Motif 1" detected by MEME analysis (120). These data indicate that these paralogs originating from a single ancestral LEA ortholog may be under similar regulation.

We also observed that LEA proteins encoded by genes in each cluster showed characteristic isoelectric points (pI); paralogs in the widely regulated group (Block 1) had moderate pI values (average 6.87), and those in the NaCl sample (Block 3) had somewhat lower values (average 5.18), but those regulated under at late trehalose treatment and early rehydration stages (Block 4) had markedly acidic (average 4.74) pI values (Figure 3b).

These observations suggests that the highly duplicated LEA protein coding genes are partitioned by functional features, possibly to allow orthologs to function in diverse cellular states.

SI REFERENCES

1. Rao, S.S., Huntley, M.H., Durand, N.C., Stamenova, E.K., Bochkov, I.D., Robinson, J.T., Sanborn, A.L., Machol, I., Omer, A.D., Lander, E.S. *et al.* (2014) A 3D map of the human genome at kilobase resolution reveals principles of chromatin looping. *Cell*, **159**, 1665-1680.

2. Watanabe, K., Imanishi, S., Akiduki, G., Cornette, R. and Okuda, T. (2016) Air-dried cells from the anhydrobiotic insect, *Polypedilum vanderplanki*, can survive long term preservation at room temperature and retain proliferation potential after rehydration. *Cryobiology*, **73**, 93-98.

3. Yamada, T.G., Hiki, Y., Hiroi, N.F., Shagimardanova, E., Gusev, O., Cornette, R., Kikawada, T. and Funahashi, A. (2020) Identification of a master transcription factor and a regulatory mechanism for desiccation tolerance in the anhydrobiotic cell line Pv11. *PLoS One*, **15**, e0230218.

4. Grapotte, M., Saraswat, M., Bessière, C., Menichelli, C., Ramilowski, J.A., Severin, J., Hayashizaki, Y., Itoh, M., Tagami, M., Murata, M. *et al.* (2021) Discovery of widespread transcription initiation at microsatellites predictable by sequence-based deep neural network. *Nat Commun.*, **12**, 3297.

5. Lassmann, T. (2015) TagDust2: a generic method to extract reads from sequencing data. *BMC Bioinformatics*, **16**, 24.

6. Marcais, G. and Kingsford, C. (2011) A fast, lock-free approach for efficient parallel counting of occurrences of k-mers. *Bioinformatics*, **27**, 764-770.

7. Vurture, G.W., Sedlazeck, F.J., Nattestad, M., Underwood, C.J., Fang, H., Gurtowski, J. and Schatz, M.C. (2017) GenomeScope: fast reference-free genome profiling from short reads. *Bioinformatics*, **33**, 2202-2204.

8. Bolger, A.M., Lohse, M. and Usadel, B. (2014) Trimmomatic: a flexible trimmer for Illumina sequence data. *Bioinformatics*, **30**, 2114-2120.

9. O'Connell, J., Schulz-Trieglaff, O., Carlson, E., Hims, M.M., Gormley, N.A. and Cox, A.J. (2015) NxTrim: optimized trimming of Illumina mate pair reads. *Bioinformatics*, **31**, 2035-2037.

10. Chikhi, R. and Medvedev, P. (2014) Informed and automated k-mer size selection for genome assembly. *Bioinformatics*, **30**, 31-37.

11. Kajitani, R., Toshimoto, K., Noguchi, H., Toyoda, A., Ogura, Y., Okuno, M., Yabana, M., Harada, M., Nagayasu, E., Maruyama, H. *et al.* (2014) Efficient de novo assembly of highly heterozygous genomes from whole-genome shotgun short reads. *Genome Res*, **24**, 1384-1395.

12. English, A.C., Richards, S., Han, Y., Wang, M., Vee, V., Qu, J., Qin, X., Muzny, D.M., Reid, J.G., Worley, K.C. *et al.* (2012) Mind the gap: upgrading genomes with Pacific Biosciences RS long-read sequencing technology. *PLoS One*, **7**, e47768.

13. Wences, A.H. and Schatz, M.C. (2015) Metassembler: merging and optimizing de novo genome assemblies. *Genome Biol*, **16**, 207.

14. Chin, C.S., Alexander, D.H., Marks, P., Klammer, A.A., Drake, J., Heiner, C., Clum, A., Copeland, A., Huddleston, J., Eichler, E.E. *et al.* (2013) Nonhybrid, finished microbial genome assemblies from long-read SMRT sequencing data. *Nat Methods*, **10**, 563-569.

15. Ye, C., Hill, C.M., Wu, S., Ruan, J. and Ma, Z.S. (2016) DBG2OLC: efficient assembly of large genomes using long erroneous reads of the third generation sequencing technologies. *Sci Rep*, **6**, 31900.

16. Gusev, O., Suetsugu, Y., Cornette, R., Kawashima, T., Logacheva, M.D., Kondrashov, A.S., Penin, A.A., Hatanaka, R., Kikuta, S., Shimura, S. *et al.* (2014) Comparative genome sequencing reveals genomic signature of extreme desiccation tolerance in the anhydrobiotic midge. *Nat Commun*, **5**, 4784.

17. Miyamoto, M., Motooka, D., Gotoh, K., Imai, T., Yoshitake, K., Goto, N., Iida, T., Yasunaga, T., Horii, T., Arakawa, K. *et al.* (2014) Performance comparison of second- and third-generation sequencers using a bacterial genome with two chromosomes. *BMC Genomics*, **15**, 699.

18. Boetzer, M., Henkel, C.V., Jansen, H.J., Butler, D. and Pirovano, W. (2011) Scaffolding pre-assembled contigs using SSPACE. *Bioinformatics*, **27**, 578-579.

19. Nadalin, F., Vezzi, F. and Policriti, A. (2012) GapFiller: a de novo assembly approach to fill the gap within paired reads. *BMC Bioinformatics*, **13 Suppl 14**, S8.

20. Dudchenko, O., Batra, S.S., Omer, A.D., Nyquist, S.K., Hoeger, M., Durand, N.C., Shamim, M.S., Machol, I., Lander, E.S., Aiden, A.P. *et al.* (2017) De novo assembly of the *Aedes aegypti* genome using Hi-C yields chromosome-length scaffolds. *Science*, **356**, 92-95.

21. Durand, N.C., Shamim, M.S., Machol, I., Rao, S.S., Huntley, M.H., Lander, E.S. and Aiden, E.L. (2016) Juicer provides a one-click system for analyzing loop-resolution Hi-C experiments. *Cell Syst*, **3**, 95-98.

22. Durand, N.C., Robinson, J.T., Shamim, M.S., Machol, I., Mesirov, J.P., Lander, E.S. and Aiden, E.L. (2016) Juicebox provides a visualization system for Hi-C contact maps with unlimited zoom. *Cell Syst*, **3**, 99-101.

23. Dudchenko, O., Shamim, M.S., Batra, S.S., Durand, N.C., Musial, N.T., Mostofa, R., Pham, M., Glenn St Hilaire, B., Yao, W., Stamenova, E. *et al.* (2018) The Juicebox Assembly Tools module facilitates *de novo* assembly of mammalian genomes with chromosome-length scaffolds for under $1000. *bioRxiv*, https://doi.org/10.1101/254797 (Preprint).

24. Gurevich, A., Saveliev, V., Vyahhi, N. and Tesler, G. (2013) QUAST: quality assessment tool for genome assemblies. *Bioinformatics*, **29**, 1072-1075.

25. Simao, F.A., Waterhouse, R.M., Ioannidis, P., Kriventseva, E.V. and Zdobnov, E.M. (2015) BUSCO: assessing genome assembly and annotation completeness with single-copy orthologs. *Bioinformatics*, **31**, 3210-3212.

26. Li, H. and Durbin, R. (2009) Fast and accurate short read alignment with Burrows-Wheeler transform. *Bioinformatics*, **25**, 1754-1760.

27. Okonechnikov, K., Conesa, A. and Garcia-Alcalde, F. (2016) Qualimap 2: advanced multi-sample quality control for high-throughput sequencing data. *Bioinformatics*, **32**, 292-294.

28. Li, H., Handsaker, B., Wysoker, A., Fennell, T., Ruan, J., Homer, N., Marth, G., Abecasis, G., Durbin, R. and Genome Project Data Processing, S. (2009) The Sequence Alignment/Map format and SAMtools. *Bioinformatics*, **25**, 2078-2079.

29. Quinlan, A.R. and Hall, I.M. (2010) BEDTools: a flexible suite of utilities for comparing genomic features. *Bioinformatics*, **26**, 841-842.

30. Kumar, S., Jones, M., Koutsovoulos, G., Clarke, M. and Blaxter, M. (2013) Blobology: exploring raw genome data for contaminants, symbionts and parasites using taxon-annotated GC-coverage plots. *Front Genet*, **4**, 237.

31. Buchfink, B., Xie, C. and Huson, D.H. (2015) Fast and sensitive protein alignment using DIAMOND. *Nat Methods*, **12**, 59-60.

32. UniProt, C. (2019) UniProt: a worldwide hub of protein knowledge. *Nucleic Acids Res*, **47**, D506-D515.

33. Smit, A. and Hubley, R. (2008-2015), http://www.repeatmasker.org.

34. Smit, A., Hubley, R. and Green, P. (2013-2015), http://www.repeatmasker.org.

35. Kim, D., Langmead, B. and Salzberg, S.L. (2015) HISAT: a fast spliced aligner with low memory requirements. *Nat Methods*, **12**, 357-360.

36. Hoff, K.J., Lange, S., Lomsadze, A., Borodovsky, M. and Stanke, M. (2016) BRAKER1: unsupervised RNA-Seq-based genome annotation with GeneMark-ET and AUGUSTUS. *Bioinformatics*, **32**, 767-769.

37. Jones, P., Binns, D., Chang, H.Y., Fraser, M., Li, W., McAnulla, C., McWilliam, H., Maslen, J., Mitchell, A., Nuka, G. *et al.* (2014) InterProScan 5: genome-scale protein function classification. *Bioinformatics*, **30**, 1236-1240.

38. Lowe, T.M. and Eddy, S.R. (1997) tRNAscan-SE: a program for improved detection of transfer RNA genes in genomic sequence. *Nucleic Acids Res*, **25**, 955-964.

39. Seemann, T. (2013). 0.6 ed, https://github.com/tseemann/barrnap.

40. Li, H. (2018) Minimap2: pairwise alignment for nucleotide sequences. *Bioinformatics*, **34**, 3094-3100.

41. Altschul, S.F., Madden, T.L., Schaffer, A.A., Zhang, J., Zhang, Z., Miller, W. and Lipman, D.J. (1997) Gapped BLAST and PSI-BLAST: a new generation of protein database search programs. *Nucleic Acids Res*, **25**, 3389-3402.

42. Wang, Y., Tang, H., Debarry, J.D., Tan, X., Li, J., Wang, X., Lee, T.H., Jin, H., Marler, B., Guo, H. *et al.* (2012) MCScanX: a toolkit for detection and evolutionary analysis of gene synteny and collinearity. *Nucleic Acids Res*, **40**, e49.

43. Bandi, V. and Gutwin, C. (2020), *Proceedings of the 46th Graphics Interface Conference on Proceedings of Graphics Interface 2020*. Canadian Human-Computer Communications Society, Waterloo, CAN.

44. Cabanettes, F. and Klopp, C. (2018) D-GENIES: dot plot large genomes in an interactive, efficient and simple way. *PeerJ*, **6**, e4958.

45. Shumate, A. and Salzberg, S.L. (2020) Liftoff: accurate mapping of gene annotations. *Bioinformatics*.

46. Duttke, S.H., Chang, M.W., Heinz, S. and Benner, C. (2019) Identification and dynamic quantification of regulatory elements using total RNA. *Genome Res*, **29**, 1836-1846.

47. Bray, N.L., Pimentel, H., Melsted, P. and Pachter, L. (2016) Near-optimal probabilistic RNA-seq quantification. *Nat Biotechnol*, **34**, 525-527.

48. Wyman, D., Balderrama-Gutierrez, G., Reese, F., Jiang, S., Rahmanian, S., Forner, S., Matheos, D., Zeng, W., Williams, B., Trout, D. *et al.* (2020) A technology-agnostic long-read analysis pipeline for transcriptome discovery and quantification. *bioRxiv*, https://doi.org/10.1101/672931 (preprint).

49. Tardaguila, M., de la Fuente, L., Marti, C., Pereira, C., Pardo-Palacios, F.J., Del Risco, H., Ferrell, M., Mellado, M., Macchietto, M., Verheggen, K. *et al.* (2018) SQANTI: extensive characterization of long-read transcript sequences for quality control in full-length transcriptome identification and quantification. *Genome Res*.

50. Wick R, Volkening J and N, L. (2018). v0.2.4 ed, https://github.com/rrwick/Porechop.

51. Bushmanova, E., Antipov, D., Lapidus, A. and Prjibelski, A.D. (2019) rnaSPAdes: a de novo transcriptome assembler and its application to RNA-Seq data. *Gigascience*, **8**.

52. Vaser, R., Sovic, I., Nagarajan, N. and Sikic, M. (2017) Fast and accurate de novo genome assembly from long uncorrected reads. *Genome Res*, **27**, 737-746.

53. ONT Research. (2019). 0.11.5 ed, https://github.com/nanoporetech/medaka.

54. Wu, T.D. and Watanabe, C.K. (2005) GMAP: a genomic mapping and alignment program for mRNA and EST sequences. *Bioinformatics*, **21**, 1859-1875.

55. Trapnell, C., Roberts, A., Goff, L., Pertea, G., Kim, D., Kelley, D.R., Pimentel, H., Salzberg, S.L., Rinn, J.L. and Pachter, L. (2012) Differential gene and transcript expression analysis of RNA-seq experiments with TopHat and Cufflinks. *Nat Protoc*, **7**, 562-578.

56. Nowell, R.W., Almeida, P., Wilson, C.G., Smith, T.P., Fontaneto, D., Crisp, A., Micklem, G., Tunnacliffe, A., Boschetti, C. and Barraclough, T.G. (2018) Comparative genomics of bdelloid rotifers: Insights from desiccating and nondesiccating species. *PLoS Biol*, **16**, e2004830.

57. Shaikhutdinov, N.M., Klink, G.V., Garushyants, S.K., Kozlova, O.S., Cherkasov, A.V., Kikawada, T., Okuda, T., Pemba, D., Deviatiiarov, R.M., Gazizova, G.R. *et al.* (2020) Population genomics of two closely related anhydrobiotic midges reveals differences in adaptation to extreme desiccation. *bioRxiv*, https://doi.org/10.1101/2020.1108.1119.255828 (Preprint).

58. Broad Institute. (2022), http://broadinstitute.github.io/picard/.

59. Li, H. (2011) A statistical framework for SNP calling, mutation discovery, association mapping and population genetical parameter estimation from sequencing data. *Bioinformatics*, **27**, 2987-2993.

60. Van der Auwera, G.A., Carneiro, M.O., Hartl, C., Poplin, R., Del Angel, G., Levy-Moonshine, A., Jordan, T., Shakir, K., Roazen, D., Thibault, J. *et al.* (2013) From FastQ data to high confidence variant calls: the Genome Analysis Toolkit best practices pipeline. *Curr Protoc Bioinformatics*, **43**, 11 10 11-11 10 33.

61. Kofler, R., Pandey, R.V. and Schlotterer, C. (2011) PoPoolation2: identifying differentiation between populations using sequencing of pooled DNA samples (Pool-Seq). *Bioinformatics*, **27**, 3435-3436.

62. Boitard, S., Kofler, R., Francoise, P., Robelin, D., Schlotterer, C. and Futschik, A. (2013) Pool-hmm: a Python program for estimating the allele frequency spectrum

and detecting selective sweeps from next generation sequencing of pooled samples. *Mol Ecol Resour*, **13**, 337-340.

63. Sedlazeck, F.J., Rescheneder, P., Smolka, M., Fang, H., Nattestad, M., von Haeseler, A. and Schatz, M.C. (2018) Accurate detection of complex structural variations using single-molecule sequencing. *Nat Methods*, **15**, 461-468.

64. Criscuolo, A. (2019) A fast alignment-free bioinformatics procedure to infer accurate distance-based phylogenetic trees from genome assemblies. *Res Ideas Outcomes*, **5**.

65. Cingolani, P., Platts, A., Wang le, L., Coon, M., Nguyen, T., Wang, L., Land, S.J., Lu, X. and Ruden, D.M. (2012) A program for annotating and predicting the effects of single nucleotide polymorphisms, SnpEff: SNPs in the genome of *Drosophila melanogaster* strain w1118; iso-2; iso-3. *Fly (Austin)*, **6**, 80-92.

66. Howe, K.L., Achuthan, P., Allen, J., Allen, J., Alvarez-Jarreta, J., Amode, M.R., Armean, I.M., Azov, A.G., Bennett, R., Bhai, J. *et al.* (2021) Ensembl 2021. *Nucleic Acids Res*, **49**, D884-D891.

67. Kim, S., Oh, M., Jung, W., Park, J., Choi, H.G. and Shin, S.C. (2017) Genome sequencing of the winged midge, *Parochlus steinenii*, from the Antarctic Peninsula. *Gigascience*, **6**, 1-8.

68. Bushnell, B. (2015), https://sourceforge.net/projects/bbmap/.

69. Kutsenko, A., Svensson, T., Nystedt, B., Lundeberg, J., Bjork, P., Sonnhammer, E., Giacomello, S., Visa, N. and Wieslander, L. (2014) The *Chironomus tentans* genome sequence and the organization of the Balbiani ring genes. *BMC Genomics*, **15**, 819.

70. Oppold, A.M., Schmidt, H., Rose, M., Hellmann, S.L., Dolze, F., Ripp, F., Weich, B., Schmidt-Ott, U., Schmidt, E., Kofler, R. *et al.* (2017) *Chironomus riparius* (Diptera) genome sequencing reveals the impact of minisatellite transposable elements on population divergence. *Mol Ecol*, **26**, 3256-3275.

71. Schmidt, H., Greshake, B., Feldmeyer, B., Hankeln, T. and Pfenninger, M. (2013) Genomic basis of ecological niche divergence among cryptic sister species of non-biting midges. *BMC Genomics*, **14**, 384.

72. Krosch, M.N. (2017) Comparative analysis of larval transcriptomes from co-occurring species of Australian *Cricotopus* (Diptera: Chironomidae). *Austral Entomol*, **56**, 451-458.

73. Krosch, M.N., Bryant, L.M. and Vink, S. (2017) Differential gene expression of Australian *Cricotopus draysoni* (Diptera: Chironomidae) populations reveals seasonal association in detoxification gene regulation. *Sci Rep*, **7**, 14263.

74. Narayanan Kutty, S., Wong, W.H., Meusemann, K., Meier, R. and Cranston, P.S. (2018) A phylogenomic analysis of Culicomorpha (Diptera) resolves the relationships among the eight constituent families. *Syst Entomol*, **43**, 434-446.

75. Mantilla, J.G., Gomes, L. and Cristancho, M.A. (2018) The differential expression of *Chironomus* spp genes as useful tools in the search for pollution biomarkers in freshwater ecosystems. *Brief Funct Genom*, **17**, 151-156.

76. Zhang, L., Yang, J., Li, H., You, J., Chatterjee, N. and Zhang, X. (2020) Development of the transcriptome for a sediment ecotoxicological model species, *Chironomus dilutus*. *Chemosphere*, **244**, 125541.

77. Beissbarth, T. and Speed, T.P. (2004) GOstat: find statistically overrepresented Gene Ontologies within a group of genes. *Bioinformatics*, **20**, 1464-1465.

78. Gurumayum, S., Jiang, P., Hao, X., Campos, T.L., Young, N.D., Korhonen, P.K., Gasser, R.B., Bork, P., Zhao, X.M., He, L.J. *et al.* (2021) OGEE v3: Online GEne Essentiality database with increased coverage of organisms and human cell lines. *Nucleic Acids Res*, **49**, D998-D1003.

79. Suyama, M., Torrents, D. and Bork, P. (2006) PAL2NAL: robust conversion of protein sequence alignments into the corresponding codon alignments. *Nucleic Acids Res*, **34**, W609-612.

80. Yang, Z. (2007) PAML 4: phylogenetic analysis by maximum likelihood. *Mol Biol Evol*, **24**, 1586-1591.

81. Li, B. and Dewey, C.N. (2011) RSEM: accurate transcript quantification from RNA-Seq data with or without a reference genome. *BMC Bioinformatics*, **12**, 323.

82. Grabherr, M.G., Haas, B.J., Yassour, M., Levin, J.Z., Thompson, D.A., Amit, I., Adiconis, X., Fan, L., Raychowdhury, R., Zeng, Q. *et al.* (2011) Full-length transcriptome assembly from RNA-Seq data without a reference genome. *Nat Biotechnol*, **29**, 644-652.

83. Love, M.I., Huber, W. and Anders, S. (2014) Moderated estimation of fold change and dispersion for RNA-seq data with DESeq2. *Genome Biol*, **15**, 550.

84. Robinson, M.D., McCarthy, D.J. and Smyth, G.K. (2010) edgeR: a Bioconductor package for differential expression analysis of digital gene expression data. *Bioinformatics*, **26**, 139-140.

85. Supek, F., Bosnjak, M., Skunca, N. and Smuc, T. (2011) REVIGO summarizes and visualizes long lists of gene ontology terms. *PLoS One*, **6**, e21800.

86. Buels, R., Yao, E., Diesh, C.M., Hayes, R.D., Munoz-Torres, M., Helt, G., Goodstein, D.M., Elsik, C.G., Lewis, S.E., Stein, L. *et al.* (2016) JBrowse: a

dynamic web platform for genome visualization and analysis. *Genome Biol*, **17**, 66.

87. Arakawa, K., Mori, K., Ikeda, K., Matsuzaki, T., Kobayashi, Y. and Tomita, M. (2003) G-language Genome Analysis Environment: a workbench for nucleotide sequence data mining. *Bioinformatics*, **19**, 305-306.

88. Arakawa, K. and Tomita, M. (2006) G-language system as a platform for large-scale analysis of high-throughput omics data. *J Pestic Sci*, **31**, 282-288.

89. Tokumoto, S., Miyata, Y., Deviatiiarov, R., Yamada, T.G., Hiki, Y., Kozlova, O., Yoshida, Y., Cornette, R., Funahashi, A., Shagimardanova, E. *et al.* (2021) Genome-wide role of HSF1 in transcriptional regulation of desiccation tolerance in the anhydrobiotic cell line, Pv11. *Int J Mol Sci*, **22**.

90. Miyata, Y., Fuse, H., Tokumoto, S., Hiki, Y., Deviatiiarov, R., Yoshida, Y., Yamada, T.G., Cornette, R., Gusev, O., Shagimardanova, E. *et al.* (2021) Cas9-mediated genome editing reveals a significant contribution of calcium signaling pathways to anhydrobiosis in Pv11 cells. *Sci Rep*, **11**, 19698.

91. Miyata, Y., Tokumoto, S., Sogame, Y., Deviatiiarov, R., Okada, J., Cornette, R., Gusev, O., Shagimardanova, E., Sakurai, M. and Kikawada, T. (2019) Identification of a novel strong promoter from the anhydrobiotic midge, *Polypedilum vanderplanki*, with conserved function in various insect cell lines. *Sci Rep*, **9**, 7004.

92. Rovira, C., Beermann, W. and Edstrom, J.E. (1993) A repetitive DNA sequence associated with the centromeres of *Chironomus pallidivittatus*. *Nucleic Acids Res*, **21**, 1775-1781.

93. Petrova, N.A., Cornette, R., Shimura, S., Gusev, O.A., Pemba, D., Kikawada, T., Zhirov, S.V. and Okuda, T. (2015) Karyotypical characteristics of two allopatric African populations of anhydrobiotic *Polypedilum* Kieffer, 1912 (Diptera, Chironomidae) originating from Nigeria and Malawi. *Comp Cytogenet*, **9**, 173-188.

94. Rosen, M. and Edstrom, J. (2000) DNA structures common for chironomid telomeres terminating with complex repeats. *Insect Mol Biol*, **9**, 341-347.

95. Lee, H., McManus, C.J., Cho, D.Y., Eaton, M., Renda, F., Somma, M.P., Cherbas, L., May, G., Powell, S., Zhang, D. *et al.* (2014) DNA copy number evolution in *Drosophila* cell lines. *Genome Biol*, **15**, R70.

96. Miller, J.R., Koren, S., Dilley, K.A., Puri, V., Brown, D.M., Harkins, D.M., Thibaud-Nissen, F., Rosen, B., Chen, X.G., Tu, Z. *et al.* (2018) Analysis of the Aedes albopictus C6/36 genome provides insight into cell line utility for viral propagation. *Gigascience*, **7**, 1-13.

97. Cornette, R., Gusev, O., Nakahara, Y., Shimura, S., Kikawada, T. and Okuda, T. (2015) Chironomid midges (Diptera, Chironomidae) show extremely small genome sizes. *Zoolog Sci*, **32**, 248-254.

98. Schmidt, E.R. (1984) Clustered and interspersed repetitive DNA sequence family of *Chironomus*. The nucleotide sequence of the Cla-elements and of various flanking sequences. *J Mol Biol*, **178**, 1-15.

99. Hankeln, T., Rohwedder, A., Weich, B. and Schmidt, E.R. (1994) Transposition of minisatellite-like DNA in *Chironomus* midges. *Genome*, **37**, 542-549.

100. Koren, S., Walenz, B.P., Berlin, K., Miller, J.R., Bergman, N.H. and Phillippy, A.M. (2017) Canu: scalable and accurate long-read assembly via adaptive k-mer weighting and repeat separation. *Genome Res*, **27**, 722-736.

101. Wyman, D. and Mortazavi, A. (2019) TranscriptClean: variant-aware correction of indels, mismatches and splice junctions in long-read transcripts. *Bioinformatics*, **35**, 340-342.

102. Mazin, P.V., Shagimardanova, E., Kozlova, O., Cherkasov, A., Sutormin, R., Stepanova, V.V., Stupnikov, A., Logacheva, M., Penin, A., Sogame, Y. *et al.* (2018) Cooption of heat shock regulatory system for anhydrobiosis in the sleeping chironomid *Polypedilum vanderplanki*. *Proc Natl Acad Sci USA*, **115**, E2477-E2486.

103. Yoshida, Y., Koutsovoulos, G., Laetsch, D.R., Stevens, L., Kumar, S., Horikawa, D.D., Ishino, K., Komine, S., Kunieda, T., Tomita, M. *et al.* (2017) Comparative genomics of the tardigrades *Hypsibius dujardini* and *Ramazzottius varieornatus*. *PLoS Biol.*, **15**, e2002266.

104. Kirkpatrick, M. (2010) How and why chromosome inversions evolve. *PLoS Biol*, **8**.

105. Sturtevant, A.H. (1926) A crossover reducer in *Drosophila melanogaster* due to inversion of a section of the third chromosome. *Biol Zentralbl*, 697-702.

106. Dobzhansky, T. (1947) Genetics of natural populations; a response of certain gene arrangements in the third chromosome of *Drosophila pseudoobscura* to natural selection. *Genetics*, **32**, 142-160.

107. Hoffmann, A.A. and Rieseberg, L.H. (2008) Revisiting the impact of inversions in evolution: from population genetic markers to drivers of adaptive shifts and speciation? *Annu Rev Ecol Evol Syst*, **39**, 21-42.

108. Noor, M.A., Grams, K.L., Bertucci, L.A. and Reiland, J. (2001) Chromosomal inversions and the reproductive isolation of species. *Proc Natl Acad Sci USA*, **98**, 12084-12088.

109. Rieseberg, L.H. (2001) Chromosomal rearrangements and speciation. *Trends Ecol Evol*, **16**, 351-358.

110. Sun, X., Liu, W., Li, R., Zhao, C., Pan, L. and Yan, C. (2021) A chromosome level genome assembly of *Propsilocerus akamusi* to understand its response to heavy metal exposure. *Mol Ecol Resour*, **21**, 1996-2012.

111. Kiknadze, I.I., Istomina, A.G., Golygina, V.V., Rubtsov, N.B. and Karamysheva, T.V. (2007) The structural peculiarities of karyotypes in species of *Propsilocerus akamusi* sibling group (Diptera: Chironomidae). *Comp Cytogenet*, **1**, 33-43.

112. Kiknadze, I.I., Wang, X. and Istomina, A.G. (2004) Karyotype of *Propsilocerus akamusi* (Tokunaga) from China (Diptera: Chironomidae). *Zootaxa*, **765**.

113. Petrova, N.A., Zelentsov, N. I., Klishko, O. K. & Chubareva, L. A. (2003) First description of polytene chromosomes, larval morphology and biology of two species of the genus *Propsilocerus* (Diptera, Chironomidae, Orthocladiinae). *Trudy Russkago entomologicheskago obshchestva (Proceedings of the Russian Entomological Society)*, **74**, 33-50.

114. Kelley, J.L., Peyton, J.T., Fiston-Lavier, A.S., Teets, N.M., Yee, M.C., Johnston, J.S., Bustamante, C.D., Lee, R.E. and Denlinger, D.L. (2014) Compact genome of the Antarctic midge is likely an adaptation to an extreme environment. *Nat Commun*, **5**, 4611.

115. Kaiser, T.S., Poehn, B., Szkiba, D., Preussner, M., Sedlazeck, F.J., Zrim, A., Neumann, T., Nguyen, L.T., Betancourt, A.J., Hummel, T. *et al.* (2016) The genomic basis of circadian and circalunar timing adaptations in a midge. *Nature*, **540**, 69-73.

116. Watanabe, M., Kikawada, T. and Okuda, T. (2003) Increase of internal ion concentration triggers trehalose synthesis associated with cryptobiosis in larvae of *Polypedilum vanderplanki*. *J Exp Biol*, **206**, 2281-2286.

117. Bus, J.S. and Gibson, J.E. (1984) Paraquat: model for oxidant-initiated toxicity. *Environ Health Perspect*, **55**, 37-46.

118. Yamada, T.G., Suetsugu, Y., Deviatiiarov, R., Gusev, O., Cornette, R., Nesmelov, A., Hiroi, N., Kikawada, T. and Funahashi, A. (2018) Transcriptome analysis of the anhydrobiotic cell line Pv11 infers the mechanism of desiccation tolerance and recovery. *Sci Rep*, **8**, 17941.

119. Ly, L.L., Yoshida, H. and Yamaguchi, M. (2013) Nuclear transcription factor Y and its roles in cellular processes related to human disease. *Am J Cancer Res*, **3**, 339-346.

120. Hatanaka, R., Gusev, O., Cornette, R., Shimura, S., Kikuta, S., Okada, J., Okuda, T. and Kikawada, T. (2015) Diversity of the expression profiles of late

embryogenesis abundant (LEA) protein encoding genes in the anhydrobiotic midge *Polypedilum vanderplanki*. *Planta*, **242**, 451-459.

ADDITIONAL FIGURES

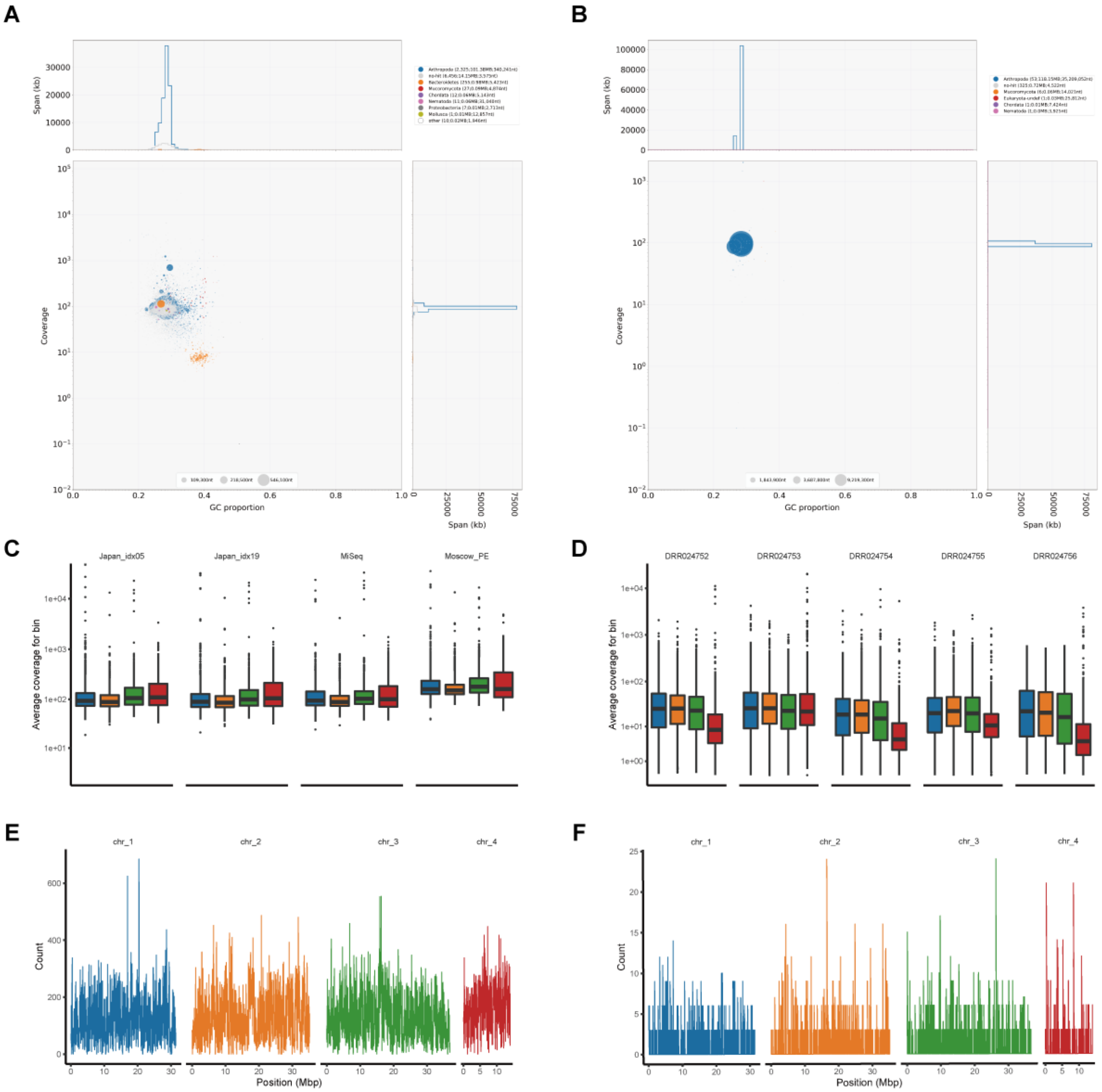

Fig. S1. Validation of the Pv5.2 genome assembly and genome-level observations. [a, b] The genome assembly obtained in this study (Pv5.2) and the Pv0.9 assembly were subjected to Blobplot analysis to identify potential contaminants. [a] Pv0.9, [b] Pv5.2. [c, d] Coverage for DNA-Seq [c] or RNA-Seq [d] data calculated for each 50kbp bins. [e, f] Number of protein-coding and non-coding genes calculated for each 50kbp windows.

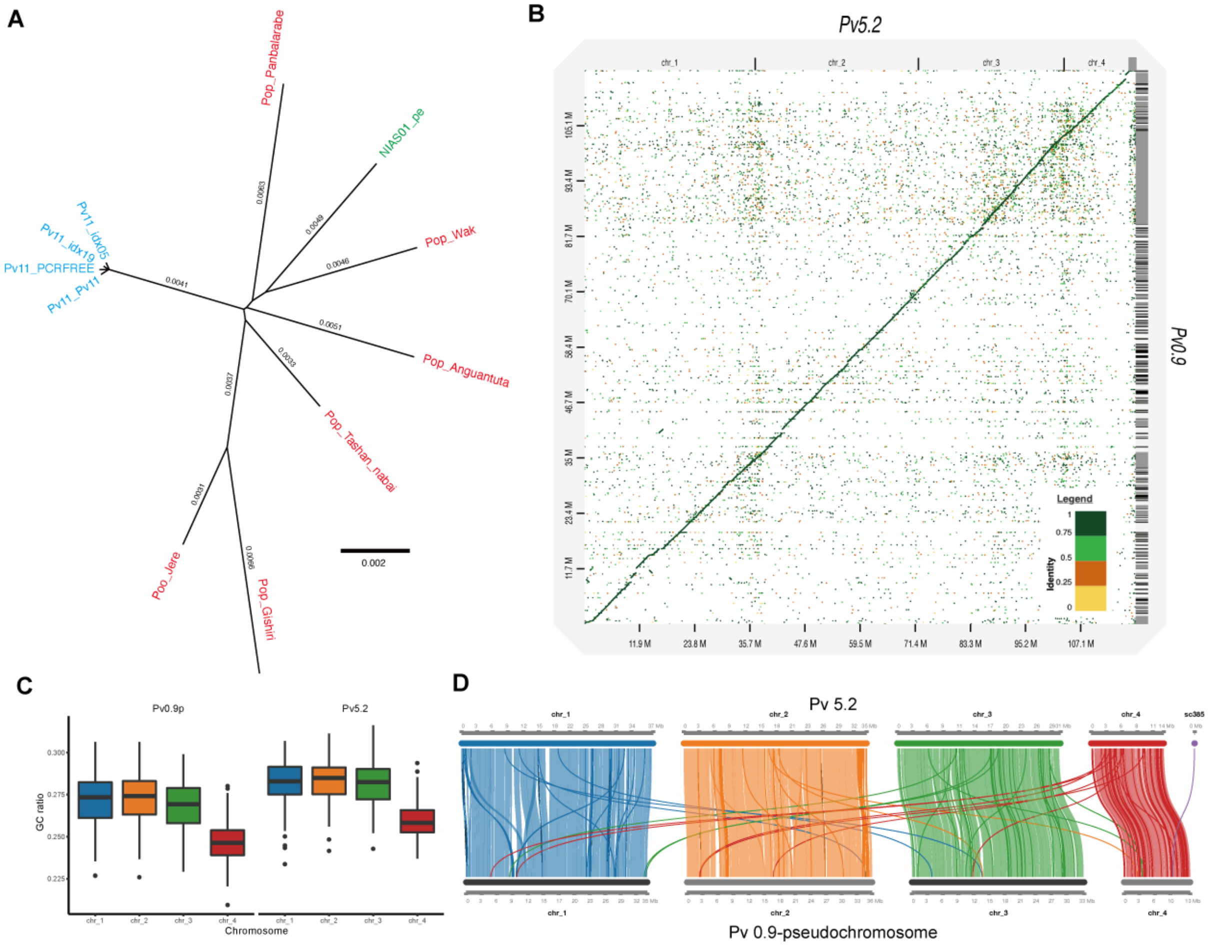

Fig. S2. Comparison between insect and Pv11 cell line genome. [a] Phylogenomic tree of wild populations (red), inbred NIAS01 strain (green) and Pv11 (blue), derived from SNV consensus sequences. Each label is designated by “Category” _” Library name”. The distance between wild populations and NIAS01 is close to that between Pv11 and others. [b] Whole genome alignment between Pv5.2 and Pv0.9 using D-Genies. The previous genome assembly Pv0.9 shows high similarity throughout the whole genome. [c] GC content of each chromosome scaffold of Pv0.9p and Pv5.2. The Pv0.9 assembly was scaffolded into “pseudochromosomes” based on the D-Genies alignment and GC ratio for each 100kbp bin was calculated with BEDtools. Colors correspond to those used in Fig 1A. [d] Detection of collinear blocks between Pv5.2 and Pv0.9p. Gene predictions were lift-over from Pv0.9 to Pv0.9p and was submitted to collinear detection with Diamond blastp and McScanX. Colinear blocks were visualized in Synvisio. Inverted collinear blocks are indicated in darker colors. Colors correspond to those used in Fig 1A.

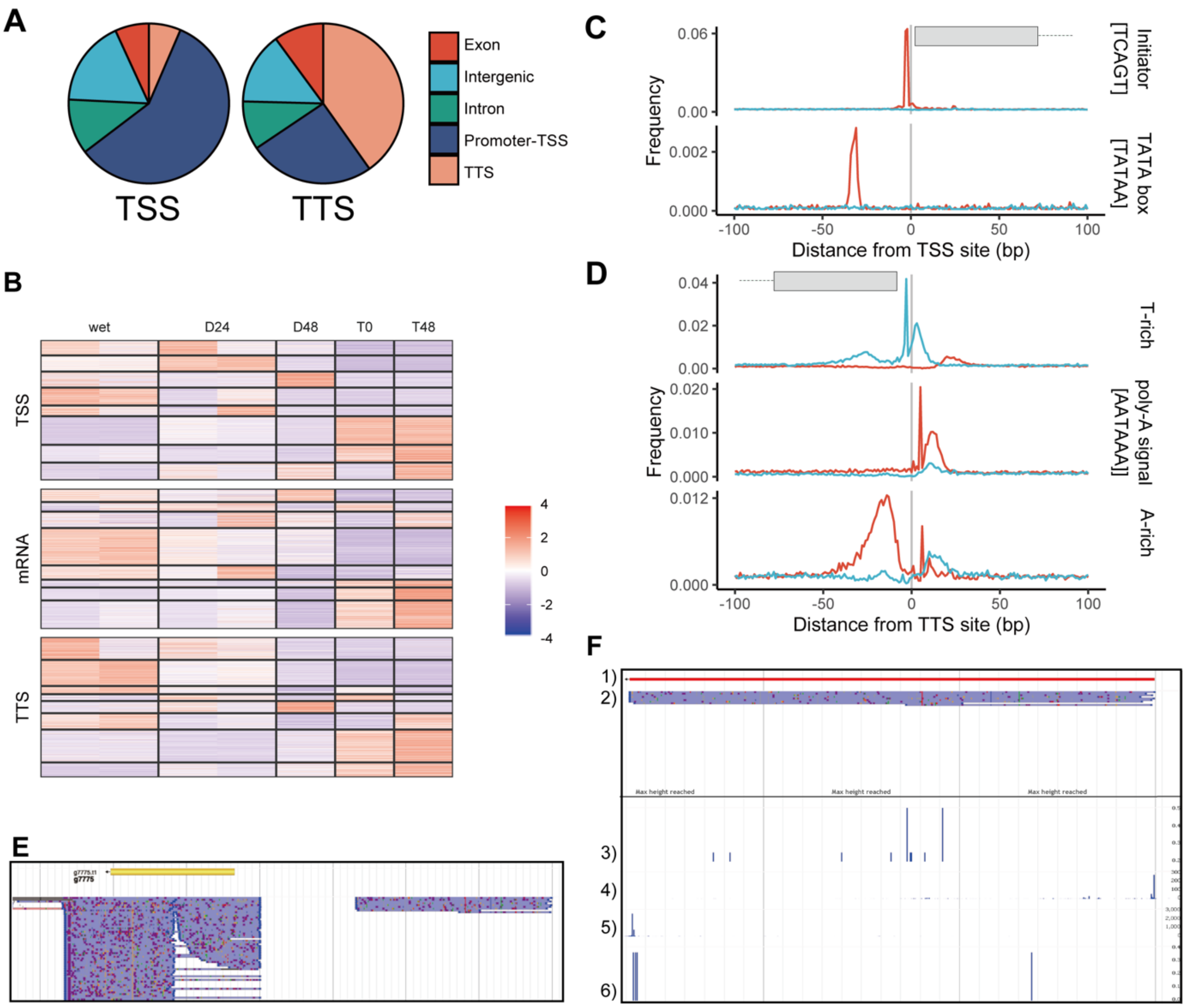

Fig. S3. CTR-Seq for simultaneous identification of TSS, TTS, and full-length transcripts. [a] Classification of identified TSS/TTS locations within the *P. vanderplanki* genome. TSS and TTS were classified with the annotatePeaks.pl utility in HOMER2. [b] Quantification of TSS, CDS, and TTS tags from CTR-Seq and visualization as a heatmap. TSS and TTS tags were normalized using UMI tags. [c] Frequencies of two core promoter motifs around TSS peaks. The gray box indicates the first exons. Red and blue lines indicate plus and negative strands, respectively. [d] Frequencies of novel motifs identified around TTS peaks. The gray box indicates the final exon. Motif #1 is the -AAUAAA- polyA signal sequence and Motifs #2 and #4 are the A-rich and U-rich domains found in close proximity to polyA signal motifs. [e] A novel non-coding gene located in the upstream region of g7775, corresponding to the 121 promoter. [f] A close-up view of the non-coding gene loci. High coverage of negative-strand mapped TSS tags and positive-strand mapped TTS tags supports a negative-strand gene: (1) The non-coding gene regions; (2) Full-length cDNA-Seq reads aligned to the genome; (3) TSS tags mapped to the positive strand; (4) TSS tags mapped to the negative strand; (5) TTS tags mapped to the positive strand; (6) TTS tags mapped to the negative strand.

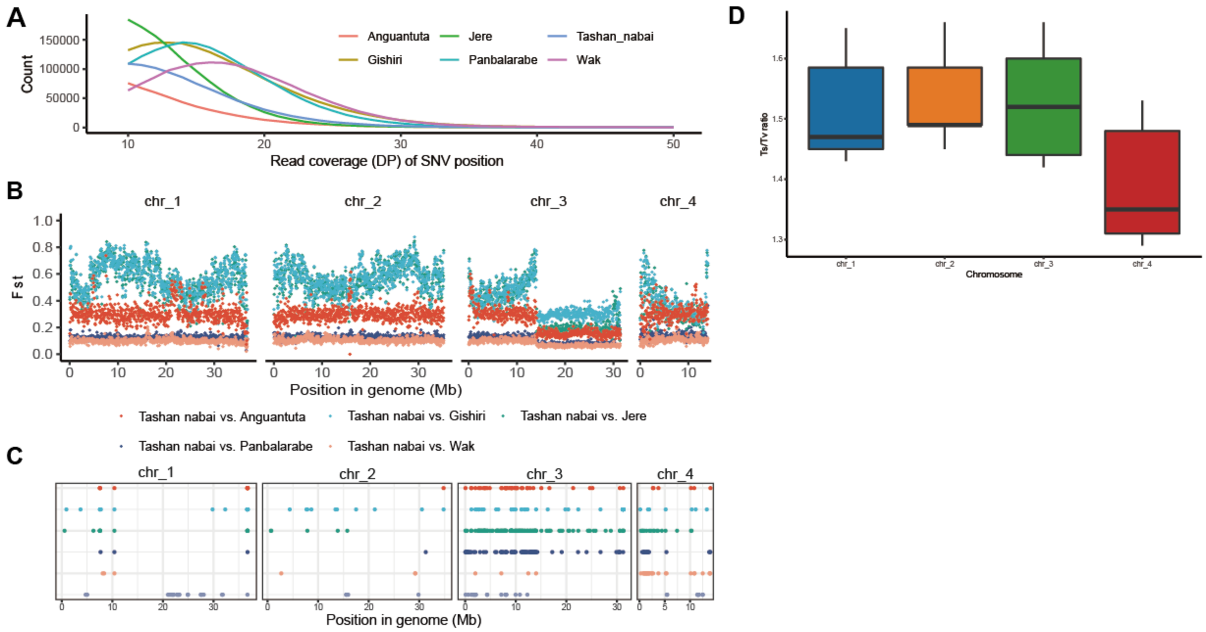

Fig. S4. Characteristic features specific of Chromosome 4 by variant analysis. Variants were detected from Pool-Seq data of 6 wild *P. vanderplanki* populations. [a] Distribution of base coverage (DP) for each SNV detected from GATK. [b] Fst values between Tashan nabai and other populations. Fst values were calculated (50kbp windows, 25kbp steps) from our Pool-Seq data of wild *P. vanderplanki* specimens and plotted against each position. [c] The locations of selection sweeps in the *P. vanderplanki* genome calculated for all six populations. [d] Single nucleotide variant Ts/Tv ratio were calculated for each chromosome in the six populations and were visualized as a boxplot. Colors correspond to those used in Fig 1A. Colors correspond to those used in Fig 1A.

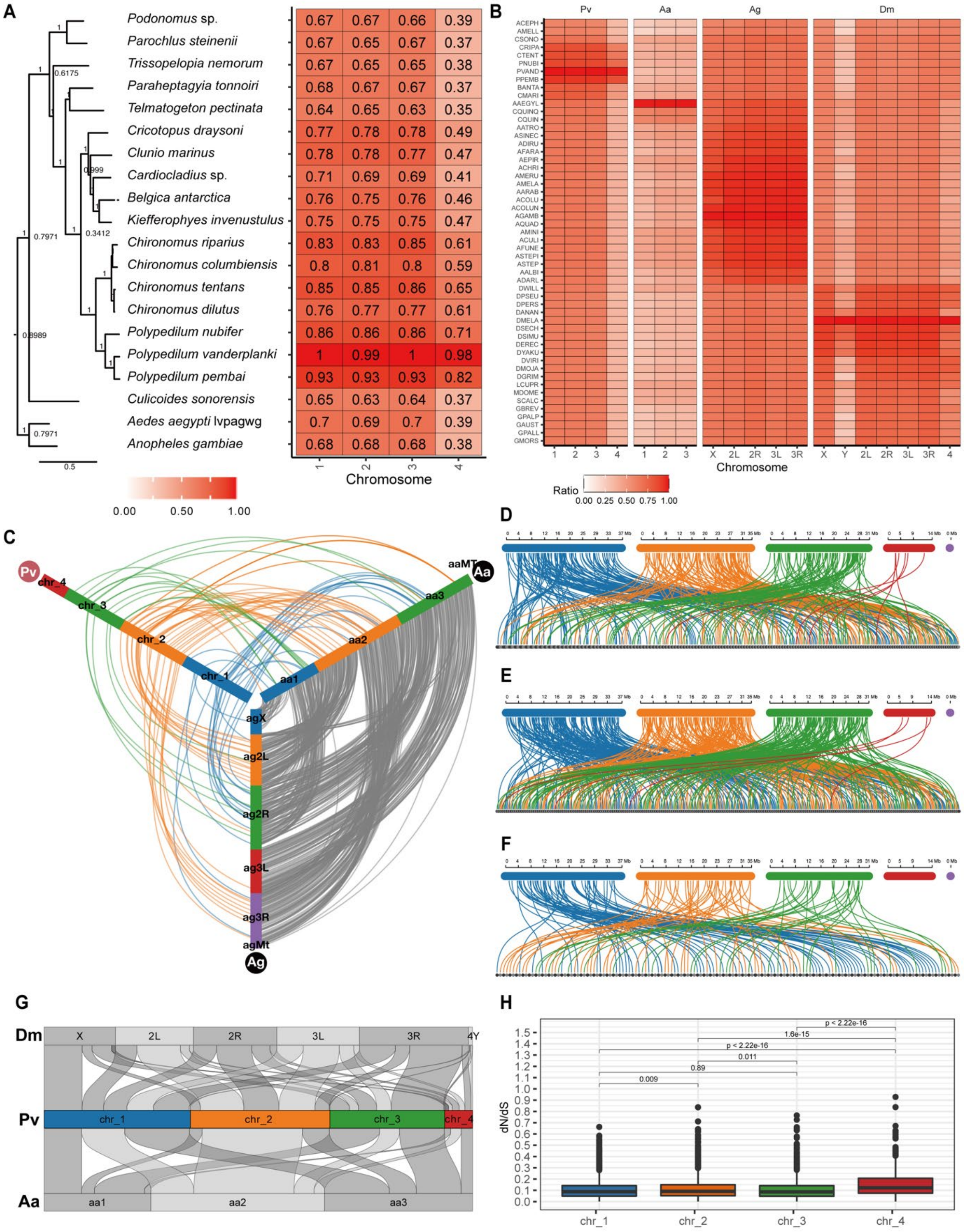

Fig. S5. Synteny analysis with chironomid species. [a] Gene conservation analysis between chironomid genomes and transcriptome assemblies. The longest isoform from each genome or isoform with the highest expression for each transcriptome assembly were subjected to the analysis conducted in Figure 2A, using *A. aegypti* and *A. gambiae* as outgroups. The

conservation ratios of *P. vanderplanki* genes (Number of genes with Diamond Blastp hits / Number of genes on chromosome) on each chromosome were visualized as a heatmap along the phylogenetic tree constructed from 666 ortholog groups. [b] Conservation analysis using *ab initio* genome prediction of all genomes from ENSEMBL, GenBank and other chironomid genomes. Genes were predicted with Braker2 and was submitted to the analysis conducted in Figure 2A. Species names are concatenated by 1st letter of the genus + 1-4th letter of the species + 1st letter of the strain (e.g., *Polypedilum vanderplanki* to PVAND). Only autosome and sex chromosomes are visualized (unplaced scaffolds are skipped). [c, d, e, f] Gene synteny analysis between [c] *P. vanderplanki* (Pv5.2), *A. aegypti* and *A. gambiae* [d] *P. vanderplanki* and *P. nubifer,* [e] *P. vanderplanki* and *C. tentans*, and [f] *P. vanderplanki* and *C. riparius*. Collinear blocks were detected by Diamond blastp and McScanX and visualized with synvisio. Chromosome colors correspond to those used in Figure 1A. [g] Location of *D. melanogaster* (Dm) and *A. aegypti* (Aa) orthologs in the *P. vanderplanki* genome determined by bidirectional best hit (BBH) method. The thickness of bars between two species indicates the percentage of genes. Genes on each chromosome were not from a single *D. melanogaster* or *A. aegypti* chromosome, rather a mixture from each chromosome suggesting Chromosome 4 is not the result of the segregation from a Dm/Aa chromosome. [h] dN/dS values were calculated for 1-to-1 orthologs between *P. vanderplanki* and *P. pembai* determined by bidirectional best hits. Colors correspond to those used in Fig 1A. *p*-values calculated from Kruskal-Wallis test are indicated.

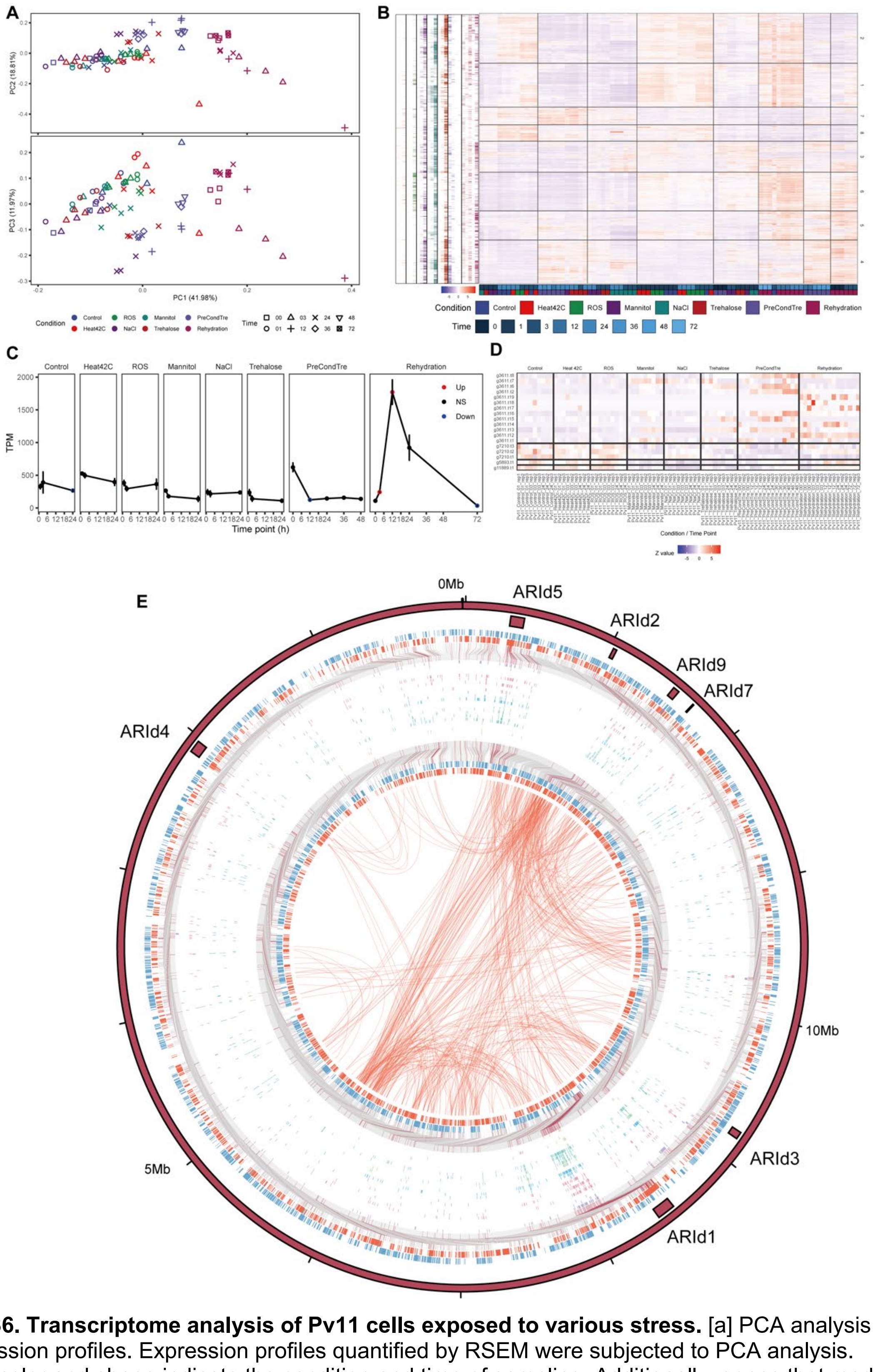

Fig. S6. Transcriptome analysis of Pv11 cells exposed to various stress. [a] PCA analysis of expression profiles. Expression profiles quantified by RSEM were subjected to PCA analysis. Each color and shape indicate the condition and time of sampling. Additionally, genes that made a marked contribution to each axis are indicated. [b] Heatmap of differentially expressed genes. The expression values of differentially expressed genes were Z-scaled and visualized as a

heatmap. The matrix on the left indicates the conditions under which the corresponding transcript is differentially expressed. The color contrast indicates the time point after treatment or condition. [c] The expression profiles of nc472, the lncRNA located upstream of g7775, were visualized with ggplot2. Error bars indicate standard deviations. [d] The expression of transcription factors: HSF (g3611) and NF-Y orthologs (NF-YA: g7210, NF-YB: g5893, NF-YC: g11889) was visualized as a heatmap using *Z*-values. [e] Circos plot of the anhydrobiosis gene-enriched Chromosome 4. The outer rim shows ARId regions, genes on the positive strand, genes on the negative strand, conditions promoting differential expression (desiccation, rehydration, trehalose, mannitol, NaCl, ROS, heat shock), genes on the positive strand, genes on the negative strand, intra-chromosome collinear blocks.

LEGENDS TO ADDITIONAL DATA

Additional Data S1. Statistics of intermediate genome assemblies.

Additional Data S2. Statistics of Hi-C read mapping (replicates marked as C1 and C2) on *P. vanderplanki* genome assembly.

Additional Data S3. Statistics of Hi-C reads (replicates marked as C1 and C2).

Additional Data S4. Candidate centromere regions.

Additional Data S5. Positions of determined TSS loci in BED format.

Additional Data S6. Positions of determined TTS loci in BED format.

Additional Data S7. Location of ARId regions.

Additional Data S8. Enrichment of repeat elements.

Additional Data S9. Statistics of variants detected from each population.

Additional Data S10. Diptera genomes used in the comparative genome analysis. *Apis mellifera* and *Atta cephalotes* were used as outgroups.

Additional Data S11. Chironomid genomes and transcriptomes used for comparative analysis. BUSCO scores that were not calculated (indicated as N.A.) for species missing TSA submissions (*C. columbiensis*, *C. dilutes*) or species with genome and gene predictions were present (*C. sonorensis*, *A. aegypti*, *A. gambiae*, *P. steinenii*, *C. marinus*, *B. antarctica*).

Additional Data S12. Statistics on the ab initio gene predictions

Additional Data S13. Number of *D. melanogaster* essential genes or BUSCO genes on each chromosome of *P. vanderplanki, A. aegypti*, and *A. gambiae*. Chromosomes with significant enrichment are indicated in red and *P. vanderplanki* Chromosome 4 in yellow.

Additional Data S14. Collated data of Chromosome 4 genes.

Additional Data S15. Enriched biological process terms of Chromosome 4 *Polypedilum*-specific genes.

Additional Data S16. Gene count table for *Polypedilum*-specific ortholog groups. Annotations for only the top 15 largest OrthoFinder ortholog groups are shown. Ionotropic receptors are indicated in red.

Additional Data S17. Top 50 transcripts with marked contribution to PC1, 2, and 3. Protein names indicated in red and blue each represent anhydrobiosis related genes and possible housekeeping genes, respectively.

Additional Data S18. Biological process terms derived from gene ontology enrichment of each DEG cluster.
